# A Super‐Capacitive Pressure Sensor with Ultrahigh Sensitivity and Wide Linear Pressure Sensing Range for Human Bio‐Signal Detection and Electronic Skin

**DOI:** 10.1002/advs.202512439

**Published:** 2025-09-12

**Authors:** Allen J. Cheng, Wenkai Chang, Zhuohan Cao, Bingnong Jiang, Yuansen Qiao, Zhao Sha, Shuai He, Chenglong Xu, Zeyad Nasa, Liao Wu, Dewei Chu, Shuhua Peng

**Affiliations:** ^1^ School of Mechanical and Manufacturing Engineering University of New South Wales Sydney NSW 2052 Australia; ^2^ School of Materials Science and Engineering University of New South Wales Sydney NSW 2052 Australia; ^3^ Micro Nano Research Facility RMIT University Melbourne VIC 3000 Australia

**Keywords:** curvy‐surface electrode, electrical double layer, hierarchical structure, linear pressure sensing range, sensitivity, super‐capacitive pressure sensor

## Abstract

Soft super‐capacitive sensors offer several advantages, including mechanical flexibility, high sensitivity, and rapid response, primarily due to the use of soft ionic elastomers and the electrical double layer (EDL) sensing mechanism. As for those sensors, achieving a broad linear pressure sensing range remains crucial, particularly when paired with a well‐defined microstructure in the electrolyte layer, which enhances sensor repeatability and facilitates quality control. In this study, a novel design strategy is proposed to simultaneously enhance sensitivity and extend the linear sensing range by integrating a hierarchical dome microstructure within the electrolyte layer and incorporating a curvilinear design in the top electrode. Specifically, the optimized sensor shows an ultrahigh sensitivity (34.79 nF kPa^−1^), a wide linear sensing range (0–800 kPa), high pressure resolution (0.1%), and stable capacitance responses for dynamic pressure even at high pressure conditions (200 kPa). Moreover, the sensor is successfully examined to perform the detection of human bio‐signals, including body movement, respiration pattern recognition, and wrist pulse detection. Additionally, the sensor demonstrates strong potential for serving as an electronic skin (e‐skin), as it enables accurate object weight estimation and object identification through machine learning techniques, achieving 100% classification accuracy.

## Graphical abstract

Incorporating a PVA/H_3_PO_4_ ionic elastomer and a hierarchical hemisphere structure within the electrolyte layer, along with a curvy top electrode, enables the super‐capacitive pressure sensor to achieve ultrahigh sensitivity (34.79 nF kPa^−1^) and a broad linear sensing range (0–800 kPa). These features highlight its strong potential for wearable electronics in human biosignal detection, health monitoring, and biomimetic e‐skin applications in soft robotics.

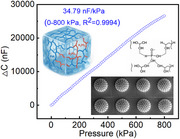

## 1 Introduction

Soft sensors and wearable electronics show the enormous application potential in soft robotics and medical devices.^[^ ^1^, ^2^, ^3^ ^]^ Specifically, flexible electronics is becoming a critical technique in human‐machine interaction,^[^ ^4^ ^]^ limb‐prosthesis,^[^ ^5^ ^]^ biomimetic e‐skin,^[^ ^6^, ^7^, ^8^ ^]^ blood pressure monitoring,^[^ ^9^, ^10^, ^11^, ^12^ ^]^ human heart rate or wrist pulse monitoring,^[^ ^13^, ^14^, ^15^ ^]^ intraocular pressure monitoring,^[^ ^16^, ^17^ ^]^ and rehabilitation training.^[^ ^18^, ^19^ ^]^ Further, detection of human body movements can also be performed with these sensors.^[^ ^20^, ^21^, ^22^ ^]^ Based on their mechanisms, wearable sensors can be categorized into piezo‐resistive,^[^ ^16^, ^23^, ^24^ ^]^ piezoelectric,^[^ ^11^, ^25^ ^]^ triboelectric,^[^ ^26^, ^27^ ^]^ and capacitive^[^ ^28^, ^29^, ^30^, ^31^ ^]^ sensors. Among these candidates, capacitive sensors demonstrate a series of merits, including simple structure, fast response, and low power consumption,^[^ ^1^ ^]^ making them an ideal and preferable candidate for wearable devices. For capacitive pressure sensors, the utmost parameters are sensitivity, linear pressure sensing range, and sensing resolution. Specifically, higher sensitivity is the prerequisite of high sensing resolution, determining the accuracy of pressure sensing, and a linear signal output simplifies the process of device calibration and data processing.^[^ ^1^ ^]^ The capacitance value of the sensor can be obtained via the equation of $C=\varepsilon \frac{A}{d}$, where ε, *A*, and *d* represent the dielectric constant, surface area of electrode and distance between two parallel electrodes, respectively. Then, the sensitivity is expressed as $k=\frac{\partial (C/C_{0})}{\partial p}$, where *C*_0_ and *p* are initial capacitance of the sensor and the applied pressure, respectively. Although linear pressure sensing range can be extended by structuring the dielectric layer, traditional parallel‐plate capacitive sensor always suffers from low sensitivity,^[^ ^32^, ^33^ ^]^ because of the limited compressibility of the dielectric layer fabricated with non‐ionic soft materials such as polydimethylsiloxane (PDMS)^[^ ^34^, ^35^ ^]^ and Ecoflex,^[^ ^36^ ^]^ affecting the sensing resolution which is crucial for wearable devices requiring precise measurement such as blood pressure monitoring. In other words, although microstructures like pyramid,^[^ ^37^ ^]^ wrinkled,^[^ ^38^ ^]^ and porous^[^ ^39^ ^]^ designs have been introduced into the dielectric layer, the sensitivity can rarely be further strengthened.

In recent years, through the application of electrical double layer (EDL) theory with soft materials containing ionic components,^[^ ^28^, ^40^, ^41^, ^42^, ^43^, ^44^, ^45^ ^]^ the sensitivity of capacitive‐type pressure sensors has been drastically elevated, denoted as super‐capacitive pressure sensors.^[^ ^13^ ^]^ Specifically, the EDL capacitance significantly incurs the capacitance change under applied pressure.^[^ ^1^, ^46^ ^]^ For a single soft ionic material, the unit area capacitance (UAC) remains constant. Thus, the value of the EDL capacitance is determined by and directly proportional to the contact area variation at the interface between electrodes and electrolyte layer, under applied pressure.^[^ ^13^ ^]^ In this case, through the structure design of the electrolyte layer, the super‐capacitive pressure sensor is able to output a linear capacitance signal within a certain pressure range. For example, a sensor using the texture structure molded by sandpaper in the electrolyte layer showed a linear capacitance signal output from 0.013 to 2063 kPa.^[^ ^47^ ^]^ In another work, the electrolyte layer was designed into a combination of dome structure and porous structure, and produced a linear capacitance signal output from 10 Pa to 400 kPa.^[^ ^48^ ^]^ All their works contributed toward the improvement in sensing performance of the super‐capacitive pressure sensor in both sensitivity and linear sensing range. However, the abovementioned textured and porous structures lack well‐defined geometries and cannot be mathematically described, which compromises the accuracy, repeatability, and consistency of signal outputs across different samples, posing a significant challenge for quality control in future practical applications.

Herein, a high‐performance super‐capacitive pressure sensor featuring a precisely defined hierarchical microstructure was designed and fabricated using 3D printing, and the sensing performance was closely related with microstructure. The poly(vinyl alcohol) and phosphoric acid (PVA/H_3_PO_4_) ionic conductive elastomer was employed to serve as the electrolyte layer for our super‐capacitive pressure sensor, to achieve a higher sensitivity under the EDL mechanism. For the linear pressure sensing range, in this work, the hierarchical hemisphere structure and a curvy surface were proposed for the electrolyte layer and the top electrode, respectively, of the super‐capacitive pressure sensor, aiming to generate more contact area variation under applied pressure. By using the high‐resolution 3D printer with high accuracy, the well‐defined hierarchical hemisphere structure was molded from a 3D printed precursor, and this structure can be mathematically expressed, helping to fabricate a consistent structure rigorously, then ensuring the repeatability and reliability of the sensor. Moreover, the curved surface of the top electrode contributes to an extended linear pressure sensing range. As a result, when both sensitivity and linear sensing range are taken into account, the super‐capacitive pressure sensor demonstrates an exceptionally high sensitivity of 34.79 nF kPa^−1^ and a wide linear pressure sensing range spanning from 0 to 800 kPa.

Based on the improved sensing performance and wide linear sensing range, the super‐capacitive pressure sensor showed its excellence in a wide variety of different perspectives. Specifically, the sensor showed high sensing resolution (0.1%), consistent capacitance output under repeated loading cycles, and reliable performance under dynamic loading conditions ranging from 0.2 to 2 Hz. Even under a preloaded high‐pressure condition of 200 kPa, the capacitance response demonstrated the sensor's ability to detect low‐pressure dynamic signals as small as 0.5 kPa at 2 Hz. As such, the sensor can successfully perform various sensing tasks, including monitoring finger, wrist, and elbow movements, as well as detecting respiration and wrist pulse, making it a strong and promising candidate for wearable device applications. Furthermore, machine learning analyses demonstrated the sensor's potential for robotic applications, such as biomimetic electronic skin, with the ability to accurately predict object weight and classify object types with 100% accuracy. These results underscore the importance of advancements in the sensing capabilities of super‐capacitive pressure sensors—particularly their enhanced sensitivity and extended linear pressure sensing range.

## 2 Results and Discussion

### 2.1 Conceptual Design of Hierarchical Electrolyte Layer and Curvy‐surface Electrode

The mechanical properties of the PVA/H_3_PO_4_ ionic elastomer were first examined to establish a fundamental understanding of the material. Mechanical stability was assessed through compression tests on a thin PVA/H_3_PO_4_ ionic elastomer film. As shown in Figure S1 (Supporting Information), cyclic loading over 100 cycles was conducted under both low (≈68 kPa) and high (≈380 kPa) pressure conditions, yielding small coefficients of variation^[^ ^13^ ^]^ of 1.21% and 1.72%, respectively, indicating stable mechanical performance. Viscoelasticity was further evaluated via compression tests, revealing a hysteresis of 19% for the unstructured thin film (Figure S2, Supporting Information).

**Figure 1:**
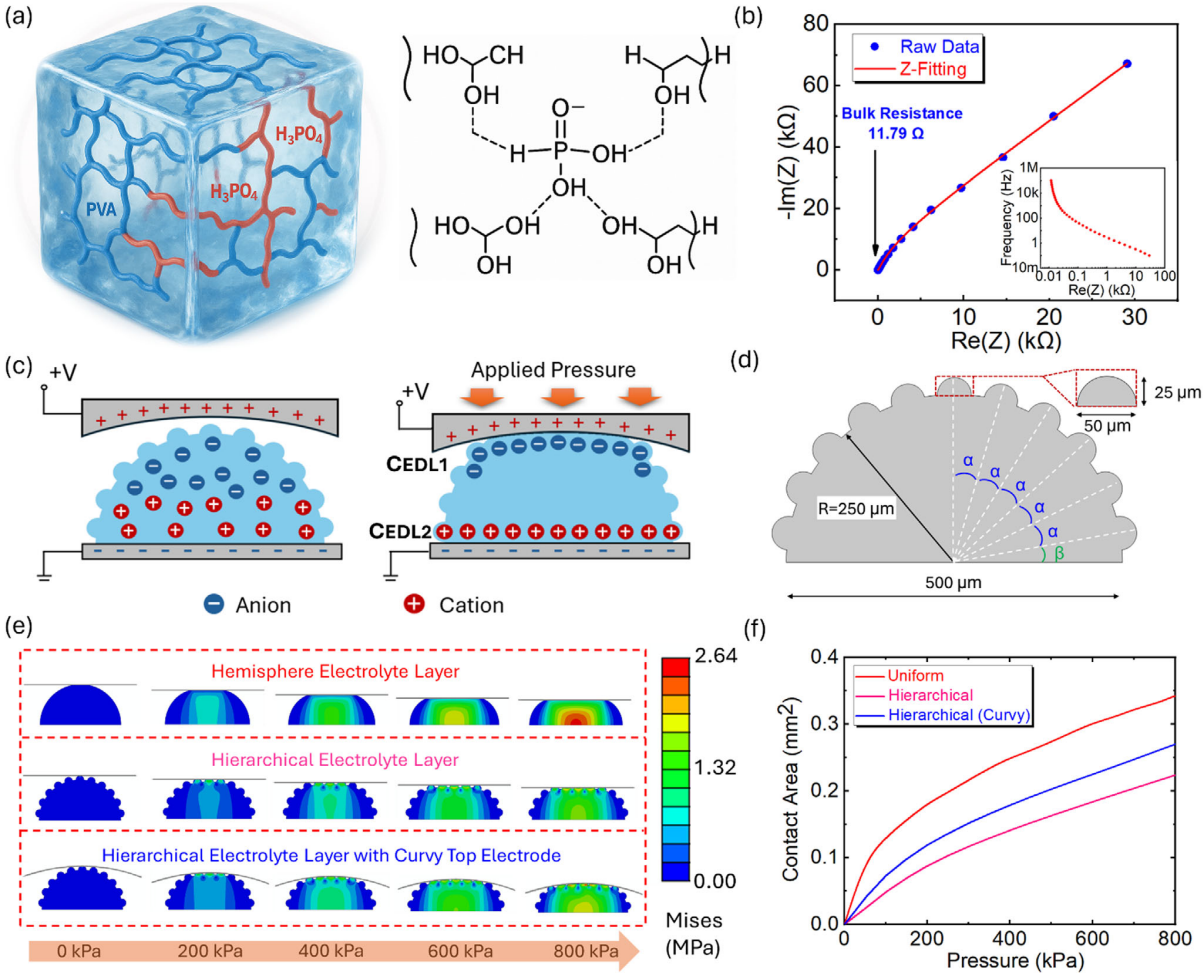
Conceptual design of a hierarchical hemisphere structure for super‐capacitive pressure sensor. a) Schematic illustration of PVA/H_3_PO_4_ ionic elastomer and its chemical formular b) Electrical properties of PVA/H_3_PO_4_ ionic elastomer. c) Schematic illustration of the EDL mechanism under applied pressure. d) 2D front view of a single unit cell for hierarchical hemisphere structure. e) Stress distribution and f) Contact area variation for three models, including uniform non‐hierarchical hemisphere with top flat surface, hierarchical hemisphere with a flat top surface, and hierarchical hemisphere with a curvy top surface.

Super‐capacitive pressure sensors with hierarchical microstructures that combine multiple feature sizes are advantageous for inducing larger changes in contact area, thereby enhancing pressure sensitivity. **Figure** **1**a shows the schematic illustration of the ionic material used for the electrolyte layer, PVA/H_3_PO_4_ ionic elastomer, and its chemical formular. The electrical properties of the ionic elastomer were characterized via electrochemical impedance spectroscopy (EIS). Briefly, an ionic elastomer thin film with a thickness of 0.43 mm was cut into a square shape of 7 × 7 mm and sandwiched by two electrodes. As shown in Figure 1b is the result of EIS measurements, showing the frequency‐dependent impedance of the ionic elastomer. The bulk resistance can be obtained, R_S_, which is 11.79 Ω. Thus, the conductivity of the ionic material, σ, can be calculated through Equation (1)

(1) $$\sigma =\frac{t}{SR_{s}}$$

where t and S denote the thickness and surface area of the specimen. Thus, the ionic conductivity of the material is 0.62 S m^−1^, providing a significant prerequisite of the EDL mechanism. Figure 1c illustrates the working principle of our super‐capacitive pressure sensors with hierarchical microstructures based on the EDL mechanism. In brief, before top electrode contacted the electrolyte layer, the sensor behaved as a traditional parallel plate capacitor. Then, when the top electrode started to touch and further compress the electrolyte layer, the sensor began to be dominated by the EDL mechanism, working as a super‐capacitive pressure sensor. Meanwhile, the polarized ions is generated at the interface between the electrolyte layer and electrodes on a thickness of nanoscale, attributed to the applied voltage which stimulates attraction between opposite charges between the electrolyte layer and electrodes.^[^ ^13^, ^41^, ^46^ ^]^ Thus, the total capacitance of a super‐capacitive pressure sensor can be expressed as C = [C_EDL1_·C_EDL2_/(C_EDL1_+C_EDL2_)]+C_E_, where C_EDL1_, C_EDL2_, and denote EDL capacitance at the top and bottom interface between electrode and electrolyte layer, respectively, and C_E_ represents the parallel‐plate capacitance. The value of CE can be neglected, as its value is 5–6 orders of magnitude smaller than EDL capacitance.^[^ ^13^, ^46^ ^]^ In this case, the capacitance for super‐capacitive pressure sensor can be re‐arranged as Equation (2)

(2) $$C=UAC\cdot \frac{A_{EDL1}\cdot A_{EDL2}}{A_{EDL1}+A_{EDL2}}\;$$

where A_EDL1_ and A_EDL2_ denote the area of contact interfaces between top, and bottom electrodes and the electrolyte layer, respectively. The equivalent circuit of the super‐capacitive pressure sensor is shown in Figure S3 (Supporting Information), providing further understanding of the super‐capacitive pressure sensor and EDL mechanism.

As shown in Figure 1d is the 2D front view of a single hierarchical hemisphere structure, and front view and top view in three‐dimension are shown in Figure S4 (Supporting Information), the diameter for large hemisphere and small hemisphere were 500 and 50 µm, respectively. In Figure 1d, α and β denote the angle between each layer and complementary angle of all layers, the values of angles α and β in this work were 16° and 10°, respectively. The hierarchical structure consisted of a small hemisphere on the top of the large hemisphere and another 5 layers of small hemispheres onto the surface of the large dome, which can be mathematically demonstrated in Figure S5 (Supporting Information). Briefly, the number of small hemispheres, N, in each layer can be summarized as Equation (3)

(3) $$N\approx \frac{2\pi \cdot \sin\left(n\alpha \right)\cdot R}{2r+d}\;$$

where n, R, r, and d denote number of small hemispheres in each layer, radius of the large hemisphere, radius of a small hemisphere, and edge distance between two small hemispheres. For the curvy‐surface top electrode, its geometry in a single unit is depicted in Figure S6 (Supporting Information). Exactly, for a single unit cell, the concave was designed with the height and width of 125 and 750 µm, respectively.

To compare the contact area variation of non‐hierarchical and hierarchical hemispheres under compression, optical microscopy was employed with a transparent rigid plate placed on top of the hemispheres. Dead weights of 800 and 2000 g were sequentially applied to the plate. As shown in Figure S7 (Supporting Information), increasing the applied weight enlarged the contact area at the interface between the hemispheres and the rigid plate. Notably, under identical compression conditions, the non‐hierarchical hemispheres exhibited a larger contact area than the hierarchical ones, suggesting that an electrolyte layer with non‐hierarchical hemispheres would produce a greater capacitance variation under the same applied pressure. This phenomenon is further supported by electrochemical impedance spectroscopy (EIS) results. The EIS experiments were conducted in two groups: one using non‐hierarchical hemispheres (Figure S8, Supporting Information) and the other using hierarchical hemispheres (Figure S9, Supporting Information). Sequential dead weights of 20, 100, 200, and 500 g were applied to the top electrodes in each group. As shown in Figures S8 and S9 (Supporting Information), higher compression reduced bulk resistance, indicating increased contact area. Under identical compression, the hierarchical hemisphere structure exhibited higher bulk resistance than the non‐hierarchical counterpart, suggesting a smaller contact area, which is consistent with the microscopy observations.

To further analyse these experimental results regarding the contact area, a finite element (FE) model was developed to simulate the contact area variation under applied pressure for a uniform non‐hierarchical hemisphere with a top flat surface, a hierarchical hemisphere with a flat top surface, and a hierarchical hemisphere with a curvy top surface. As shown in Figure 1e, the computational simulation results elucidated the stress distribution for these three models. To be specific, the uniform non‐hierarchical hemisphere suffered from the highest stress concentration at the central part under the same applied pressure. For the hierarchical structure, there was a higher pressure concentration inside the structure when the flat top surface was replaced by the curvy one. In other words, a larger deformation of the hierarchical structure resulted from the usage of a curvy surface. These phenomena can be reflected by the contact area variation under applied pressure in Figure 1f. Precisely and correspondingly, the result of uniform non‐hierarchical hemisphere produced the largest variation in contact area under applied pressure. However, there was a strong non‐linear relationship between applied pressure and contact area. Then, as the uniform non‐hierarchical hemisphere was substituted by the hierarchical structure, there was a better linearity between applied pressure and contact area, although the contact area variation was comparatively smaller. To ensure compensation for the contact area, a curvy surface can be employed, generating more contact area under applied pressure for the hierarchical structure, as shown in Figure 1f, potentially dedicated to improving sensitivity but simultaneously maintaining the linear pressure sensing range of the super‐capacitive pressure sensor. The compression of various electrolyte layers in computational simulation was recorded and shown in Videos S1–S3 (Supporting Information).

### 2.2 Preparation and Microscopy of the Curvy‐Surface Electrode and Electrolyte Layers

**Figure 2:**
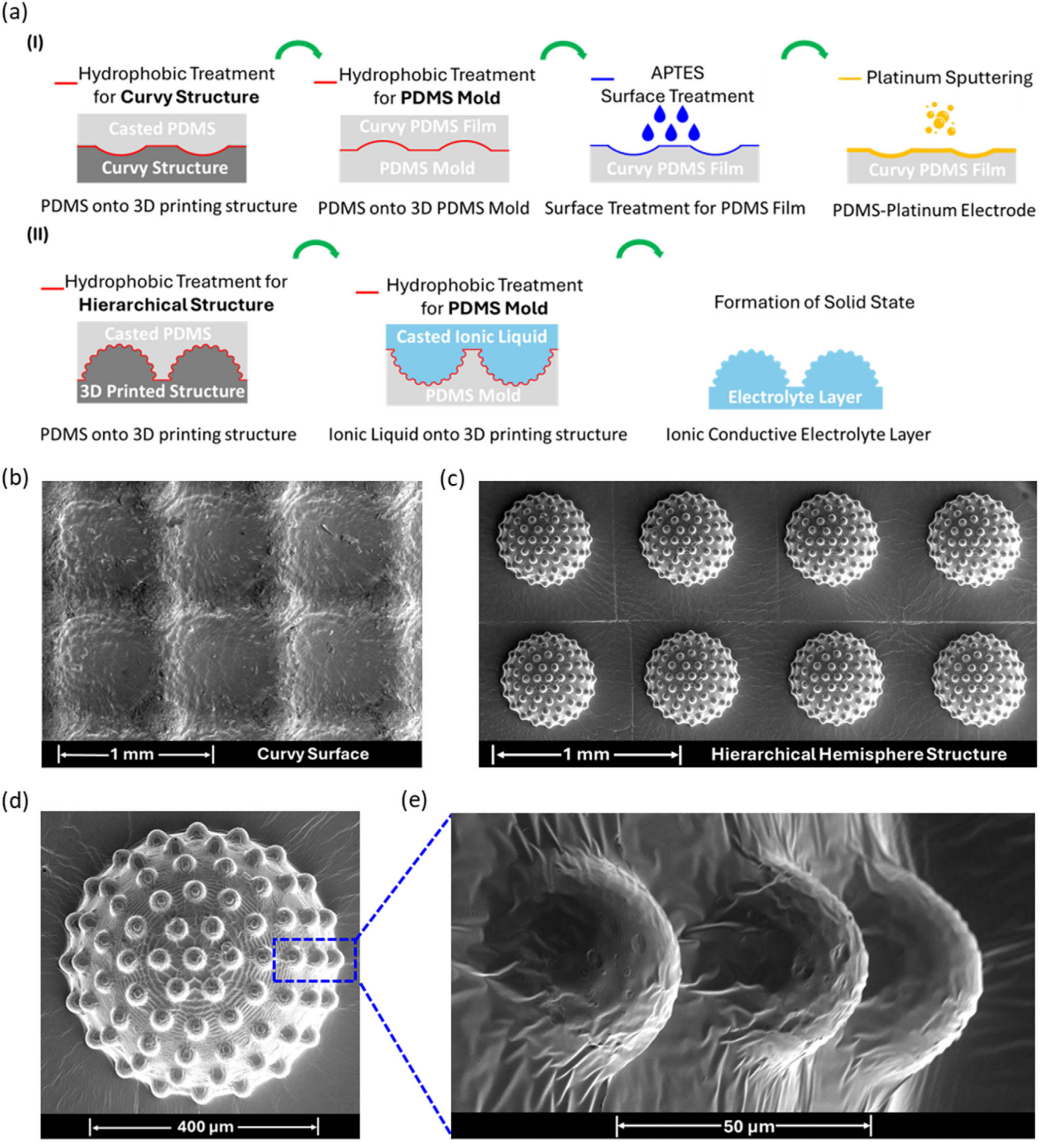
Preparation and microscopy of curvy‐surface electrode and electrolyte layer. a) Manufacturing procedure for curvy‐surface electrode and electrolyte layer with hierarchical hemisphere array. b) SEM image of curvy‐surface electrode. c) SEM image of hierarchical hemispheres. d) SEM image of a single unit of hierarchical hemisphere. e) Zoomed‐up image of small hemispheres within hierarchical hemisphere structure.

High‐resolution 3D printing was employed to fabricate a precisely defined hierarchical microstructure for pressure sensors. **Figure** **2**a demonstrates the manufacturing process of the hierarchical hemisphere structure in 20 × 20 array (Figure S10, Supporting Information) and curvy‐surface electrode. Briefly, both the hierarchical microstructure and the curvy‐surface electrode were manufactured through PDMS molds based on the 3D printed precursors. Details of the procedure were presented in Sections 4.1 and 4.2. Generally, gold was coated onto the PDMS curvy surface, serving as the top electrode. Figure 2b is the scanning electron microscopy (SEM) characterization for the top electrode, illustrating the curvy surface, aiming to generate more contact area with the electrolyte layer. In addition, the profile scanning of the curvy surface for the electrode is shown in Figure S11 (Supporting Information). The SEM image and scanning profile for uniform non‐hierarchical hemispheres are shown in Figure S12 (Supporting Information) and Figure S13 (Supporting Information), respectively. As shown in Figure 2c, the hierarchical hemispherical structure was fabricated with high accuracy. Figure 2d presents a single unit of the hierarchical hemisphere, while Figure 2e provides a magnified view of the smaller hemispheres. The base hemisphere has a diameter of 500 µm, and the perched hemisphere measures 50 µm, both dimensions aligning well with our original design. The scanning profile of the hierarchical hemisphere structure is provided in Figure S14 (Supporting Information).

### 2.3 Characterization of the Super‐Capacitive Pressure Sensors

**Figure 3:**
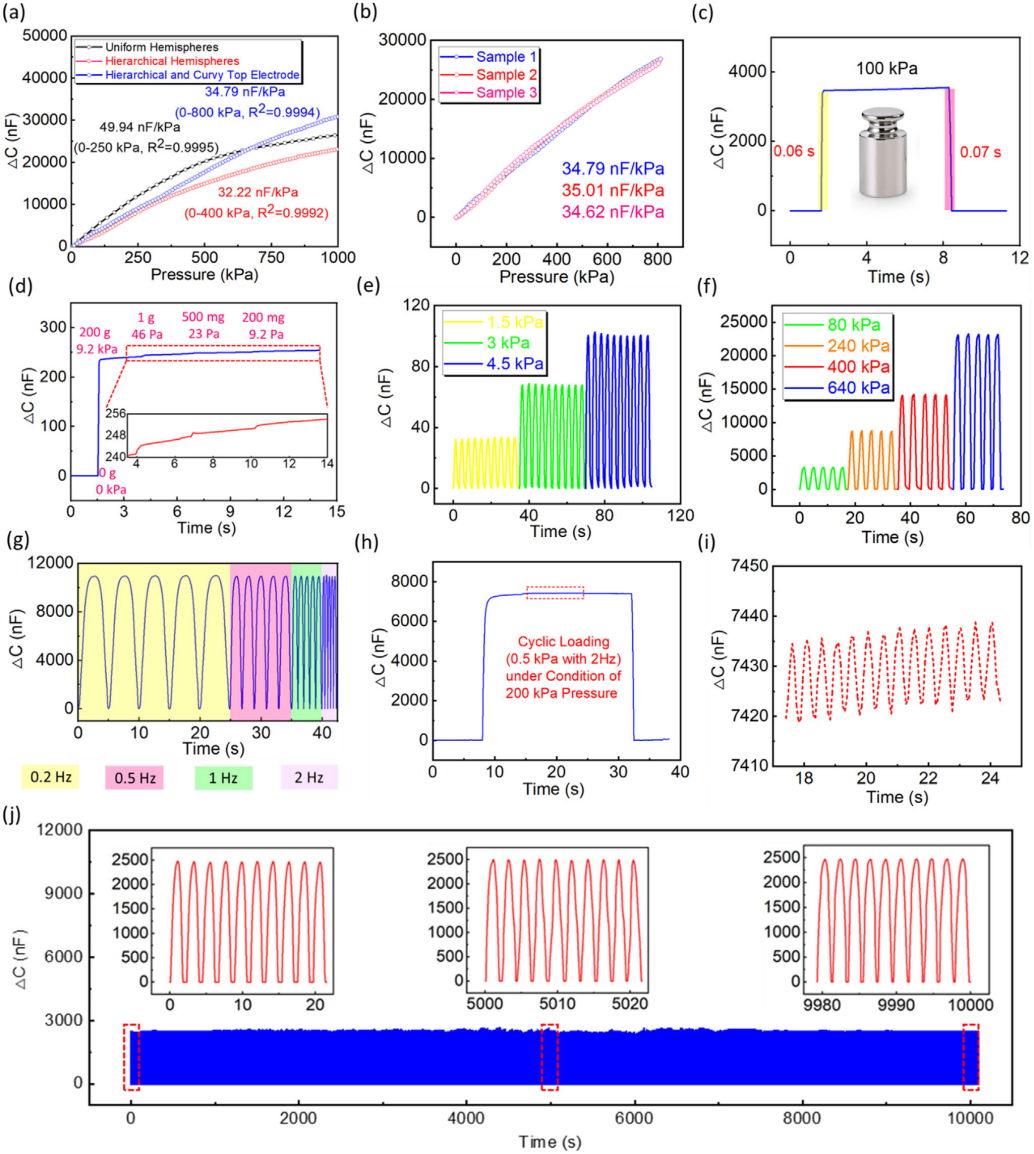
Characterization of super‐capacitive pressure sensors. a) Sensitivity and linear sensing range for three different super‐capacitive pressure sensors. b) Consistency with three samples of super‐capacitive sensor with an identical design. c) Response time of super‐capacitive pressure sensor. d) Resolution of super‐capacitive pressure sensor. Capacitance response of super‐capacitive pressure sensor under e) small pressure conditions and f) comparative large pressure conditions. g) Capacitance signal output of super‐capacitive pressure sensor under pressure of 280 kPa with mechanical frequencies from 0.2 to 2 Hz. h) Signal output of super‐capacitive pressure sensor of cyclic loading of 0.5 kPa with mechanical frequency of 2 Hz under pre‐pressure of 200 kPa. i) Illustration of the capacitance signal of cyclic loading of 0.5 kPa with a mechanical frequency of 2 Hz. j) Stability and repeatability of super‐capacitive pressure sensor under loading‐unloading conditions for more than 5000 cycles spanning over 10 000 s.

The sensing performance is a prerequisite for potential applications, including but not limited to sensitivity and linear sensing range. Above‐mentioned in computational simulation, the relationship between changes of contact area and applied pressure varies depending on micro‐structure designs. Herein, **Figure** **3**a shows the corresponding experimental results with respect to the sensitivity and linear sensing range of three super‐capacitive pressure sensors. The traditional method to define the sensitivity is heavily subject to the initial capacitance, whose value varies by a large margin in accordance with various designs. Therefore, the sensitivity is expressed by the increment in capacitance per unit of pressure, which is nF kPa^−1^, eliminating the uncertainty of sensitivity brought by initial capacitance. In a sensing system with linear response, this new approach is more scientific in showing the sensitivity of the super‐capacitive pressure sensor by reading the capacitance variation between the corresponding points of pressure. The super‐capacitive pressure sensor using a uniform non‐hierarchical hemisphere structure with the flat‐surface top electrode shows a noticeable nonlinear capacitance response under applied pressure, although it showed the highest sensitivity (49.94 nF kPa^−1^) within the pressure range from 0 to 250 kPa. The hierarchical hemisphere structure in the electrolyte layer endowed the super‐capacitive pressure sensor with a wider linear pressure sensing range (0–400 kPa), with the flat‐surface top electrode. However, the sensitivity value experienced a decrease to 32.22 kPa, aligning with the tendency computed by the FE model, because of a reduction in EDL contact area. To alleviate this problem, guided by the FE model, the top electrode of the sensor was transformed into a curvy surface from a flat one, partially compensating for the decrease in EDL contact area under applied pressure. As a result, the sensor incorporating a hierarchical hemispherical structure in the electrolyte layer, combined with a curved‐surface top electrode, exhibited a twofold increase in the linear pressure sensing range (0–800 kPa) compared to its counterpart with a flat‐surface top electrode. Additionally, it achieved a higher sensitivity (34.79 nF kPa^−1^) and the most significant linearity factor (27976) among the three sensor configurations. The linear factor was defined by sensitivity multiplied by linear sensing range. The comparison of the sensitivity and linear sensing range of our work and other works is shown in Figure S15 (Supporting Information).^[^ ^6^, ^15^, ^28^, ^31^, ^42^, ^49^, ^50^, ^51^ ^]^ Figure 3b shows the excellent consistency among three samples of the sensor using a hierarchical hemisphere electrolyte layer and curvy‐surface top electrode. Quantitatively, these samples showed a sensitivity of 34.79, 35.01, and 34.62 nF kPa^−1^, with a mean value of 34.81 ± 0.16, and the variation coefficient was 0.4% only. The definition and calculation method for the variation coefficient were included in our previous work.^[^ ^13^ ^]^

Besides sensitivity and linear pressure sensing range, other features of the sensor are of vital importance as well. As shown in Figure 3c, the sensor exhibited rapid response and relaxation times of 0.06 and 0.07 s, respectively, under a 100 kPa load applied via dead weight. This indicates that the hierarchical microstructure undergoes rapid deformation under pressure and promptly recovers upon pressure release, demonstrating the structural robustness and resilience of the PVA/H_3_PO_4_ ionic material. Fast response is an essential requirement for wearable applications such as wrist pulse monitoring and other physiological signal detection. Another crucial feature of the sensor is sensing resolution, especially under high pre‐load conditions. Since the sensitivity of our sensor did not drop within a broad pressure range, the subtle change in pressure can be detected. According to Figure 3d, small pressures, 46, 23, and 9.2 Pa were added subsequently, under a high‐pressure situation of 9.2 kPa, the corresponding capacitance response can be observed obviously. Thus, the sensor was endowed with a sensing resolution of 0.1%, which is promising for flexible electronics such as e‐skin or medical garments. Together with sensitivity and linear sensing range, parameters of response time and pressure resolution were compared between this work and other works, as summarized in Table S1 (Supporting Information).

To examine the stability of our sensor, repeated loads were gradually added, and the capacitance response was presented. As shown in Figure 3e and Figure S16 (Supporting Information), within a lower pressure range, the sensor gave a steady capacitance signal output of the 10‐time repeated loads of 1.5, 3, and 4.5 kPa, with the coefficient variation of 1.33%, 0.32% and 1.07%, respectively. In addition, capacitance response at higher pressure conditions was examined as well in a similar manner. To be specific, the sensor showed a stable capacitance signal under the 5‐time cyclic loading under the pressures of 80, 240, 400, and 640 kPa, reflected by Figure 3f and Figure S17 (Supporting Information), with the variation coefficient of 0.42%, 0.16%, 1.93% and 0.13%, respectively. These results reflected that the super‐capacitive sensor was able to generate a stabilized signal response under each pressure condition and verified that the sensor had a linear capacitance signal output with increasing pressure, in agreement with the results in Figure 3a,b. Under health monitoring, providing a steady capacitance signal output with a certain mechanical frequency is essential, such as human pulse and heart monitoring. In this research, the sensor experienced dynamic loads of 60 and 280 kPa, as illustrated in Figure S18 (Supporting Information) and Figure 3g, from mechanical frequencies from 0.2 to 2 Hz. The results demonstrated the stable capacitance signal output of the super‐capacitive pressure sensor under dynamic pressures with various mechanical frequencies, aiming at fulfilling the requirements of the potential application of the sensor for medical purposes. Considering the super‐capacitive pressure sensor was endowed with both high pressure‐resolution and an exceptionally stable capacitance signal output, and some specific monitoring may require a pre‐pressure like pulse detection, the sensor is expected to detect the small dynamic pressure under a high load condition. Therefore, as elaborated in Figure 3h, a cyclic loading of 0.5 kPa with the mechanical frequency of 2 Hz was exerted under a pre‐pressure of 200 kPa, and a clear signal of 0.5 kPa cyclic loading under 2 Hz can be output by the sensor in terms of Figure 3i. The mean value of the capacitance variation for this cyclic loading is 15.6 ± 0.79 nF, with a coefficient variation of 5.09%. Hence, the sensor detected the dynamic pressure of ≈3 orders smaller than its pre‐pressure, which is a considerable merit of the super‐capacitive pressure sensor. For wearable applications, repeatability and durability of the super‐capacitive sensor are of vital significance to ensure the reliability of the sensing device. According to Figure 3j, a cyclic loading onto the sensor of ≈70 kPa with more than 5000 cycles spanning over 10 000 s was presented, and the result showed that there is no visible drift of the capacitance signal during the 5000‐cycle whole loading‐unloading process, indicating a good reliability of the super‐capacitive pressure sensor. Ten cycles were picked up at the start, midway and the end of the cyclic loading, their values of variation coefficient were 0.31%, 0.30% and 0.12%, respectively, and their mean values are shown in Figure S19 (Supporting Information).

### 2.4 Detection of Human Bio‐Signals

**Figure 4:**
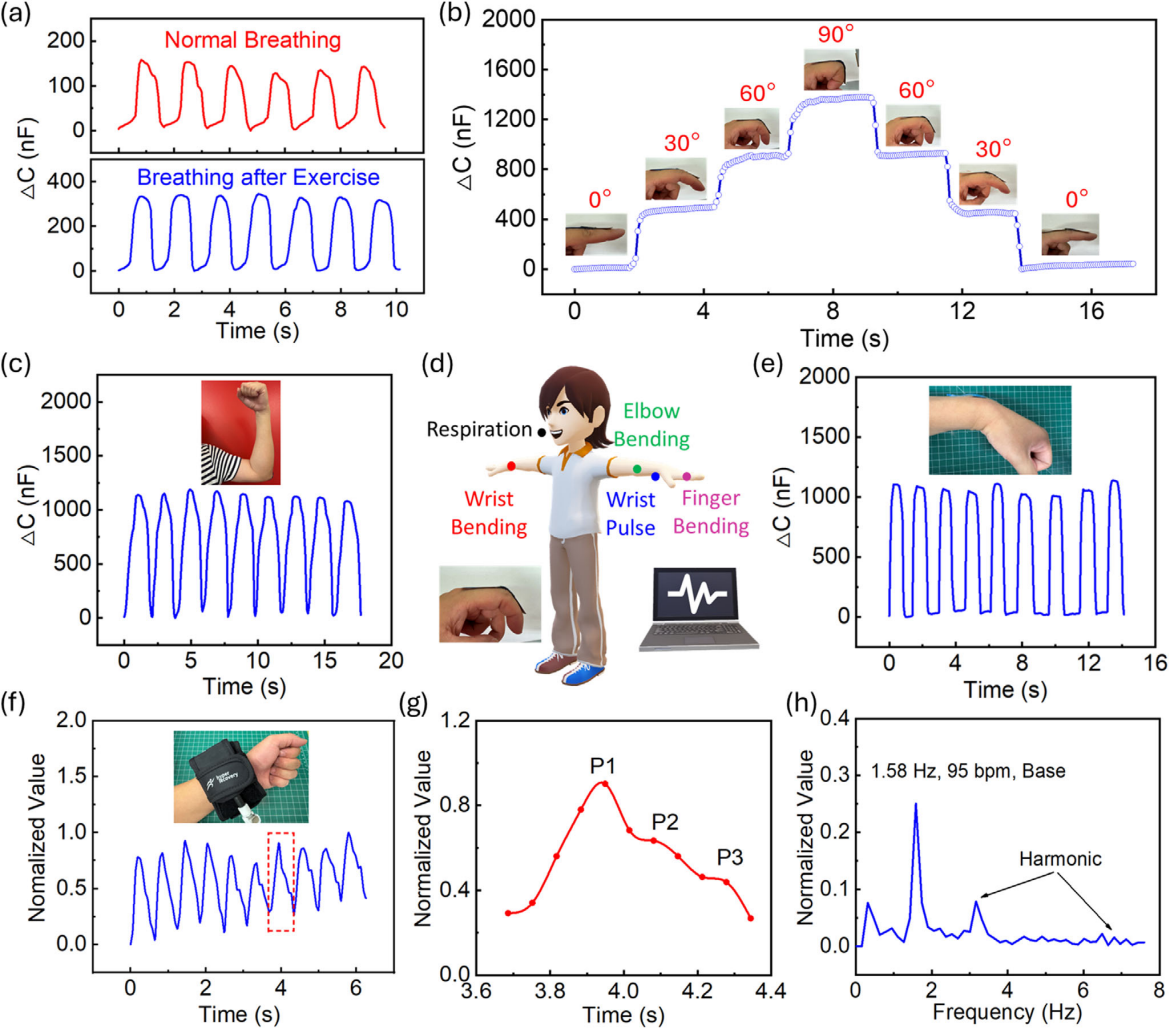
Human body bio‐signal recognition, detection, and monitoring by super‐capacitive pressure sensor. a) Recognition of human respiration pattern. b) Detection of finger bending in different angles in a round turn. c) Detection of elbow bending. d) Schematic demonstration of human body signal collections. e) Detection of wrist bending. f) Monitoring of human wrist pulse. g) Analysis of human wrist pulse. h) FFT analysis on wrist pulse and heart rate.

Because of the exceptional performance of the super‐capacitive pressure sensor granted by the hierarchical electrolyte layer and curvy top electrode, it was used to perform human body bio‐signal detection and monitoring. Specifically, the sensor was shown successfully to recognize the respiration pattern, monitor the human wrist pulse, and detect the finger bending in various angles, elbow bending, and wrist bending, according to **Figure** **4**d. Breathing is one of the most important human activities, and it varies under various conditions, such as after exercise, bronchitis, and asthma. As shown in Figure 4a, the breathing patterns of normal breathing and after mild exercise were differentiated by the sensor. As observed, the signal of the breathing pattern after a mild exercise is stronger and more intensive than that of normal breathing, consistent with the human physical characteristics before and after the exercise. Besides, bio‐signals of human body movements are rather considerable for post‐operation rehabilitation, the check the normal function of human body parts. As presented in Figure 4b, the sensor was mounted onto the middle phalanx of the index finger, and bio‐signals of the finger bending were detected by the sensor. Clear capacitance variations can be observed as the finger experienced bending angles of 30°, 60°, and 90° in a round turn, showing the detecting accuracy and consistency of finger joint movements. Moreover, bio‐signals of arm bending and wrist movements were shown in Figure 4c,e, respectively, indicating that the human physiological signals were converted into electrical signals via capacitance values by the super‐capacitive pressure sensor. Apart from these, human wrist pulse is able to show the artery stiffness, cardiovascular health condition, and other features of a human, such as age.^[^ ^52^ ^]^ In this case, the monitoring and analysis of wrist pulse is promising to provide a diagnosis of these diseases from a non‐invasive perspective. Herein, as shown in Figure 4f, the super‐capacitive sensor showed the strong capability of human wrist pulse monitoring, and a certain pre‐pressure was supplied by the medical cuff onto the sensor, and three peaks, P_1_, P_2_ and P_3_, representing percussion wave, tidal wave and diastolic wave, respectively, can be detected by the sensor, according to the spline curve in Figure 4g. The percussion wave means the sum of the ejected wave and reflected wave from human hand, whereas the tidal wave denotes the difference of the peak of the reflected wave from the lower body and end‐diastolic pressure.^[^ ^52^ ^]^ Human age can be estimated through the Radial Augmentation Index, AIr, defined by the value of P_2_/P_1_.^[^ ^52^ ^]^ According to the value in Figure 4g, the AIr value is ≈0.57, indicating the wrist pulse is from a human at the age of early 30s, fitting with the real age of the participant. Thus, the capacitance signal from the sensor can precisely reflect the specific information through monitoring the human wrist pulse. In addition, through the Fourier transform (FFT) analysis, the heart rate can be obtained through the wrist pulse monitoring, at 95 bpm. The recording of wrist pulse monitoring was shown in Video S4 (Supporting Information). In this case, the sensor provides enormous potential for the application of wearable real time point‐of‐care health monitoring device.

### 2.5 Machine Learning for Weight Prediction and Identification of Different Objects

**Figure 5:**
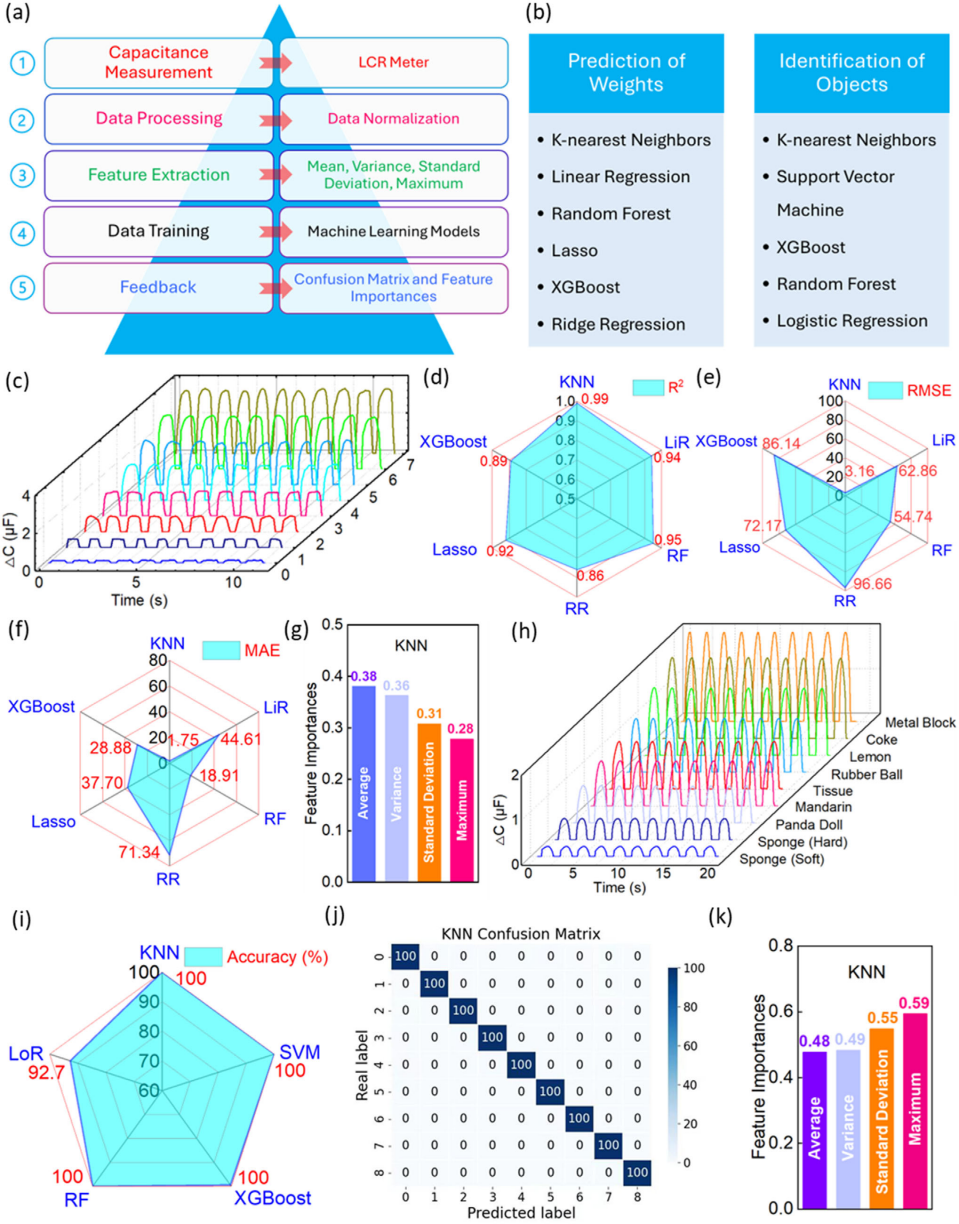
Demonstration of machine learning for weight prediction and identification of various objects. a) Methodology for machine learning. b) Machine learning models for weight prediction and identification of various objects. c) Capacitance signal output for grasping objects with different weights of 10, 20, 50, 100, 200, 300, 500, and 1000 g. d) R^2^ e) RMSE f) MAE values for weight prediction using six different machine learning models. g) Feature importances for weight prediction of KNN model. h) Capacitance signal output for touching and pressing various objects. i) Accuracy of object identification using five different machine learning models. j) Confusion matrix and k) feature importances of KNN model for object identification.

Apart from wearable applications, the super‐capacitive pressure sensor showed enormous potential serving as a biomimetic e‐skin as well. The sensor was examined to predict the weight and recognize the type of different objects, with the analysis by machine learning.^[^ ^53^ ^]^ **Figure** **5**a elaborates the overall methodology for machine learning, including capacitance acquisition, data processing, feature extraction, data training and feedback. Several machine learning models were utilized for weight prediction and object identification, as illustrated in Figure 5b. Regression algorithms were applied for weight prediction, while classification algorithms were used to identify different objects. Four statistical features, including mean, variance, standard deviation, and maximum value, were extracted for the analysis.

Weight prediction is one of the key functions of biomimetic e‐skin. When grabbing an object, a human is unable to perceive the actual weight of the object without a sensing device. Herein, a super‐capacitive pressure sensor can provide feedback on the weight of the object, serving as an e‐skin. The sensor was mounted on a human finger. Figure 5c and Figure S20 (Supporting Information) show the capacitance response of grasping objects with different weights (Figure S21, Supporting Information), 10, 20, 50, 100, 200, 300, 500 and 1000 g, by human hands, with corresponding variation coefficient of the peak value were 1.7%, 1.2%, 1.0%, 1.6%, 1.7%, 2.0%, 1.2% and 1.3%, respectively. Regularly, stronger capacitance responses could be observed as the weight of objects increased. Regression models, including k‐nearest neighbours (KNN), linear regression (LiR), random forest (RF), least absolute shrinkage and selection operator (Lasso), extreme gradient boosting (XGBoost), and ridge regression (RR) were used to perform the weight prediction with the capacitance signal output by the super‐capacitive pressure sensor. Finally, three parameters, coefficient of determination (R^2^), root‐mean‐square error (RMSE), and mean absolute error (MAE) were brought to evaluate the machine learning results. The equations of these three parameters were provided in Note S1 (Supporting Information). As shown in Figure 5d, among these models, KNN showed the largest value (0.99) in R^2^, indicating the highest accuracy for weight prediction of objects. The values of RMSE and MAE were 3.16 and 1.75, as displayed in Figure 5e,f, respectively. Within these 6 models, KNN showed the lowest figures in both RMSE and MAE, reflecting the best accuracy for weight prediction of objects. Therefore, KNN is the best model because of its highest accuracy for weight prediction of various objects. Figure 5g shows the feature importances scores for the KNN model, the largest contribution was average value (0.38), whereas the maximum value showed the smallest contribution (0.28) in this model, indicating that the average value was considerably emphasized at the first priority, followed by another two features, variation (0.36) and standard deviation (0.31). The feature importances scores of other regression models for weight prediction were shown in Figure S22 (Supporting Information), and summarization was provided in Table S2 (Supporting Information) for all the models. In this case, considering the feasibility of weight perception and high accuracy of object recognition with deep learning, the super‐capacitive pressure sensor had the potential to serve as the biomimetic e‐skin for soft robotics, facilitating the progress of artificial intelligence.

Biomimetic e‐skin is preferable to have the capability to recognize different objects, like human skin. Thus, in this work, the super‐capacitive pressure sensor served as the e‐skin to distinguish different objects. Generally, human skin shows a certain elasticity. In this case, a soft spring ≈200 N m^−1^ was attached to the sensor to mimic the elasticity of human skin, and the experimental setup is shown in Figure S23 (Supporting Information). The touching and pressing toward the pressure were controlled by a moving distance of 10 mm. Specifically, if the sensor touched and then pressed a hard object, the deformation would be undertaken by the spring attached to the sensor, while the soft object experienced the majority of the deformation under applied pressure, showing a similar situation to human skin when touch and press objects in a variety of softness or hardness. Nine different objects, denoted as 0–8 in sequence, as shown in Figure S24 (Supporting Information), were distinguished by the super‐capacitive pressure sensor in accordance with the capacitance output signals, as shown in Figure 5h and Figure S25 (Supporting Information). There are 100 sets of data for each category of the object. For example, touching and pressing toward a can of coke by the sensor had a smaller signal output than that of a metal block, because of the curvy surface of a can of coke, which only had partial contact with the sensor. Also, there were smaller capacitance signal outputs when the sensor touched and pressed the softer objects. Fix models for classification were used to perform the analysis on object recognition. Apart from KNN, RF, and XGBoost, another two models, support vector machine (SVM) and logistic regression (LoR), were introduced for classification analysis. As shown in Figure 5i, models of KNN, SVM, XGBoost, and RF showed an accuracy of 100% in recognition of various objects, and LoR model gave an accuracy of 92.7%. Another parameter to evaluate the classification result in machine learning is F1 score.^[^ ^54^ ^]^ Specifically, except LoR model, whose F1 score is 93.1%, another four models provided a F1 score of 100%, according to Figure S26 (Supporting Information). In this case, both the accuracy and F1 score shows KNN, SVM, XGBoost, and RF models were able to distinguish the results of capacitance response generated by super‐capacitive pressure sensor when touching and pressing these objects, in 100%. For example, the accuracy for object recognition using the KNN model was expressed in a confusion matrix, as shown in Figure 5j, which is 100%. The confusion matrix for the rest of the four models was shown in Figure S27 (Supporting Information). Figure 5k shows the feature importances of the KNN model for object recognition. To be specific, the maximum value counted for the largest importance (0.59), while the average value (0.48) was the least significant one, within the KNN model. The significant importances for another four models were visually supplied in Figure S28 (Supporting Information), and their values for all models were summarized in Table S3 (Supporting Information).

## 3 Conclusion 

In this work, a super‐capacitive pressure sensor was developed featuring a hierarchical hemispherical electrolyte layer and a curved‐surface top electrode, which facilitated significant and consistent changes in the EDL area under applied pressure. This design achieved ultrahigh sensitivity (34.79 nF kPa^−1^), a broad linear sensing range (0–800 kPa), and high pressure resolution (0.1%). Besides, the sensor was endowed with excellent stability, repeatability, and durability, reflecting the robustness of the sensor brought by the structured electrolyte layer and top electrode. Moreover, the sensor was examined for the effectiveness of human body bio‐signal detections, such as body movements, respiration pattern recognition, and wrist pulse monitoring, showing the great potential in wearable applications for medical purposes, such as post‐surgical rehabilitation and point‐of‐care real‐time monitoring. Significantly, the sensor was employed to perform as an e‐skin, demonstrating its capabilities to perceive the weight and recognize the category of the object through deep learning. Thus, the super‐capacitive pressure sensor possessed a bright prospect in various aspects, including medicine and robotics.

## 4 Experiments and Computational Modelling

### Procedure of Hydrophobic and Hydrophilic Surface Treatment

Prior to the hydrophobic surface treatment, plasma bonding for 7 min is required to clear the surface of the sample. Then hydrophobic surface treatment was performed with 1H,1H,2H,2H‐Perfluorooctyltriethoxysilane (500 µL) through chemical vapor deposition (CVD). The chemical was put into the vacuum chamber together with the samples. Then the vacuum chamber would be placed in the oven at a temperature of 55 °C for 7 h, before being put at room temperature for 12 h to achieve a better deposition of the chemical onto samples’ surface.

For the hydrophilic surface treatment, (3‐Aminopropyl)triethoxysilane (APTES) was applied to complete the surface treatment for the sample. Briefly, the sample was dipped into an aqueous solution consisting of 100 µL of the ATPES chemical and 20 mL of the deionized water for 24 h.

### Preparation of the PDMS Mold

To begin with, the hierarchical hemisphere structure (Figure S29, Supporting Information) was 3‐D printed by a 3D laser lithography system (Nanoscribe) with high precision. Then the 3D printed precursor experienced the CVD hydrophobic surface treatment before casting the PDMS (Sylgard 184 silicon elastomer) mixture onto the structure. The PDMS mixture was obtained by a combination of PDMS and the cure agent (Dow Coming Co. Ltd. (Australia)), with a weight ratio of 10:1. Subsequently, once the degassing process was completed, to remove the bubbles inside the PDMS mixture, the casted PDMS was delivered into oven at 80 °C for 5 h for curing before peeling the micro‐structure off the mold.

A curvy‐surface structure was 3D printed (Formlabs Form 3) as the first step, and a PDMS mixture was cast onto the curvy‐surface structure after the CVD hydrophobic surface treatment.

### Preparation of PVA/H3PO4 Ionic Aqueous Solution

PVA (Mw of 89000‐98.000 g mol^−1^) and H_3_PO_4_ (85% w/w) were purchased from Sigma–Aldrich (Australia) and Chem‐Supply (Australia), respectively. To obtain the PVA/H_3_PO_4_ ionic aqueous solution, PVA was dissolved in deionized water with a weight ratio of 1:9 by magnetic stirring with 600 rpm, at a temperature of 70 °C for 2 h. After cooling down, H_3_PO_4_ was slowly dropped into the PVA aqueous to form the PVA/H_3_PO_4_ ionic aqueous solution with an ionic concentration of 10%.

### Fabrication of Electrolyte Layer, Curvy‐surface Top Electrode, and Assembly of the Sensor

Once the CVD hydrophobic surface treatment for the PDMS mold was done, PVA/H_3_PO_4_ ionic aqueous solution was cast onto the mold and placed in a dehumidifying environment for 36 h, and lastly peeled off the structured ionic elastomer from the mold, serving as the electrolyte layer. For the preparation of the curvy‐surface top electrode, the PDMS was poured onto the 3D printed mold whose surface had experienced the CVD hydrophobic surface treatment, before putting it into the oven 80 °C for 5 h for curing, before peeling the PDMS film with a curvy surface. Subsequently, the PDMS film with a curvy surface underwent the hydrophilic surface treatment to strengthen the bonding before sputtering gold by Quorum Q300T Sputter, with a thickness of 50 nm, serving as the top electrode of super‐capacitive pressure sensor. The sensor was assembled under an optical microscope to ensure the alignment and consistency between electrodes and the electrolyte layer. Then the sensor was encapsulated by PDMS thin films with a thickness of 100 µm.

### Characterization and Demonstration of Super‐capacitive Pressure Sensor

Contact area observation, scanning profile, and micro‐structures observation were performed by Digital OLYMPUS DigRetina 16, OLYMPUS DXS510, and FEI Nova Nano SEM450, respectively. For the sensitivity characterization, a compression spring with the constant of 16 755 N m^−1^ was used to supplied pressure (Figure S30, Supporting Information), and compression load was supplied by Mark‐10 ESM 303 with the force gauge ranging from 0–1000 N, while capacitance was measured and collected by an LCR meter (KEYSIGHT Technology, E4980A) under Cs‐Rs mode, under a 1 V applied voltage and testing frequence of 1 kHz. Dynamic loads different mechanical frequencies, and cyclic loads were supplied by a ZABER linear stage. All the measurements with respect to capacitance were measured by the same LCR meter.

### Finite Element Models

FE analysis^[^ ^55^, ^56^ ^]^ was conducted using 2D plane‐strain models to investigate the mechanical behaviour, with particular focus on the variation in contact area at the interface between the top electrode and the electrolyte layer under applied pressure in different scenarios. The model configuration, featuring a hierarchical electrolyte layer in contact with a curved electrode surface, is shown in Figure S31 (Supporting Information). The hierarchical electrolyte is modelled as an elastic material with a Young's modulus of 4.5 MPa and a Poisson's ratio of 0.4,^[^ ^13^ ^]^ and discretized using 2D plane‐strain elements. The curved electrode is modelled as an analytical rigid surface.^[^ ^57^ ^]^ To replicate the experimental setup described in Section 4.5, a displacement‐controlled compression was applied to the top electrode in the negative y‐direction. The bottom boundary of the hierarchical hemisphere (i.e., the electrolyte layer) was constrained in the y‐direction only, as shown in Figure S31 (Supporting Information). A frictionless, hard contact interaction^[^ ^57^ ^]^ was defined to simulate the contacting interface between the hierarchical electrolyte and the electrode. All simulations were carried out using ABAQUS 2021, with stress and deformation results analysed accordingly.^[^ ^57^ ^]^

## Conflict of Interest

The authors declare no conflict of interest.

## Supporting information



Supporting Information

Supplemental Video 1

Supplemental Video 2

Supplemental Video 3

Supplemental Video 4

Supporting Information

## Data Availability

The data that support the findings of this study are available from the corresponding author upon reasonable request.
