# Supplementary material for: A Super‐Capacitive Pressure Sensor with Ultrahigh Sensitivity and Wide Linear Pressure Sensing Range for Human Bio‐Signal Detection and Electronic Skin
Source: Adv Sci (Weinh). 2025 Sep 12;12(45):e12439. doi: 10.1002/advs.202512439 (PMC12677675; doi:10.1002/advs.202512439)
Supplement: Supplementary file 1 — Supporting Information [file ADVS-12-e12439-s003.docx]

Supporting Information

**A super-capacitive pressure sensor with ultrahigh sensitivity and wide linear pressure sensing range for human bio-signal detection and electronic skin**

Allen J. Cheng^a^, Wenkai Chang^a^*, Zhuohan Cao^a^, Bingnong Jiang^a^, Yuansen Qiao^a^, Zhao Sha^a^, Shuai He^a^, Chenglong Xu^c^, Zeyad Nasa^c^, Liao Wu^a^, Dewei Chu^b^, Shuhua Peng^a^*

^a^ School of Mechanical and Manufacturing Engineering, University of New South Wales, Sydney, NSW 2052, Australia

^b^ School of Materials Science and Engineering, University of New South Wales, Sydney, NSW 2052, Australia

^c^ Micro Nano Research Facility, RMIT University, Melbourne, VIC 3000, Australia

Emails: [shuhua.peng@unsw.edu.au](mailto:shuhua.peng@unsw.edu.au), wenkai.chang@unsw.edu.au

**Figure S1**


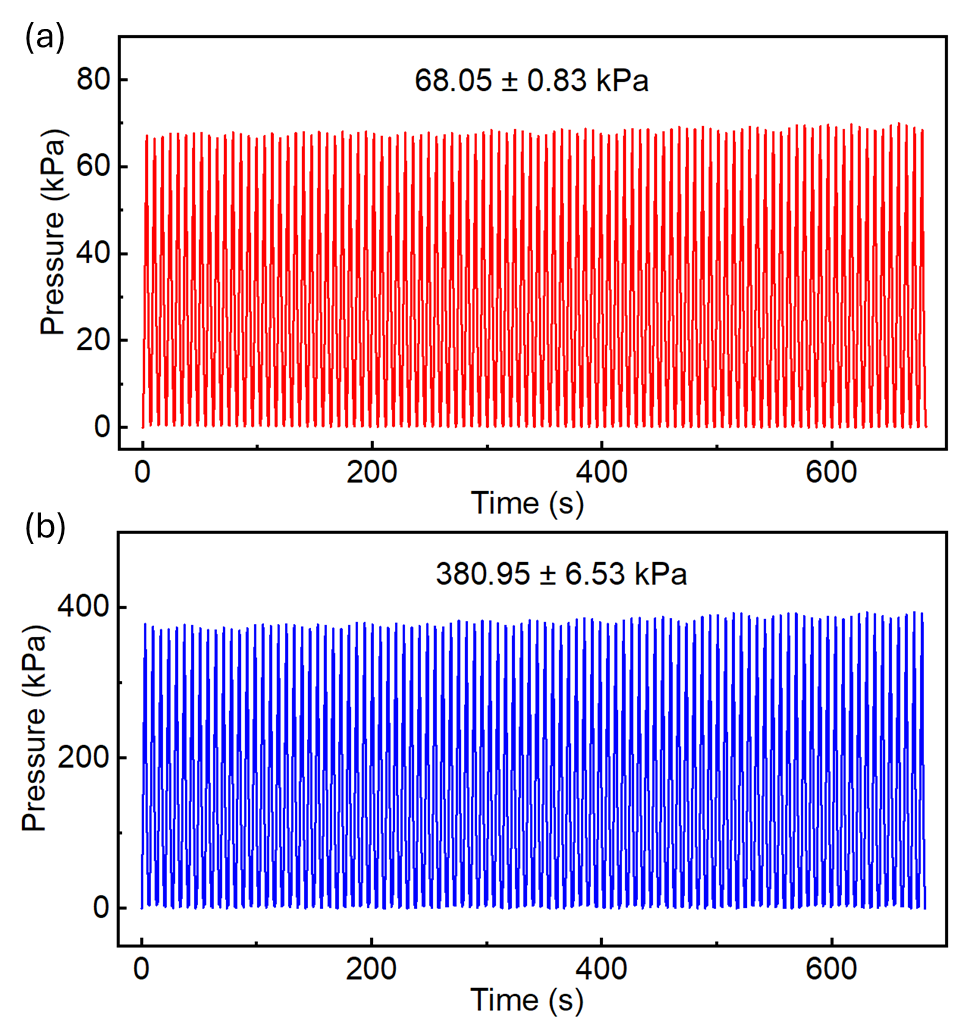


**Figure S1.** Demonstration of mechanical stability of PVA/H3PO4 ionic elastomer thin film under different pressure conditions: (a) 68kPa (b) 380 kPa

**Figure S2**


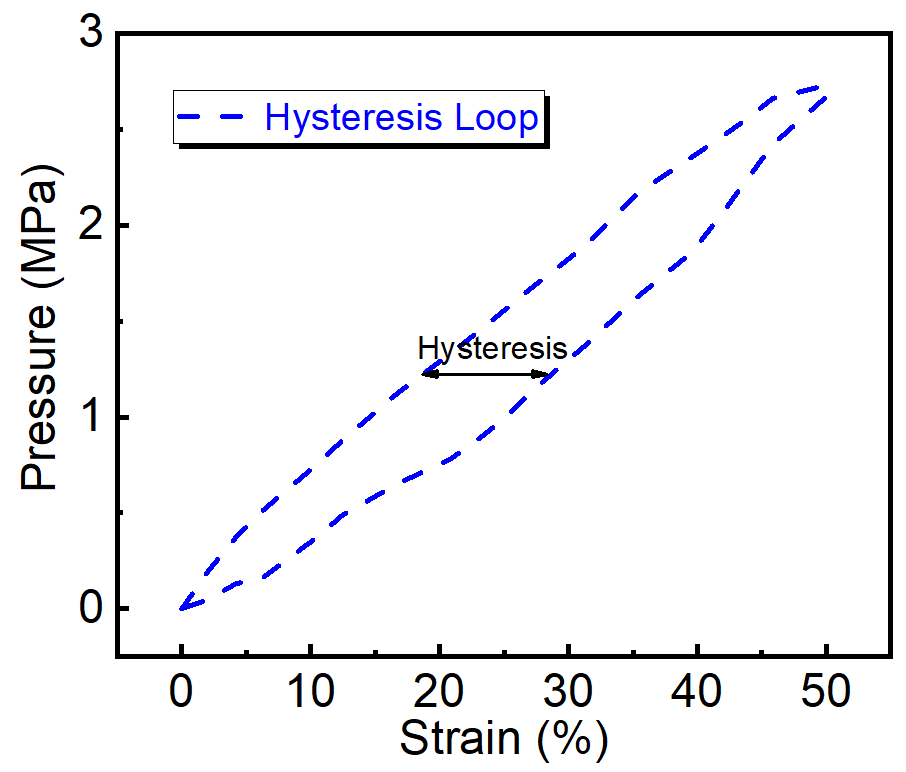


**Figure S2.** Viscoelasticity of PVA/H_3_PO_4_ ionic elastomer through hysteresis loop.

**Figure S3**


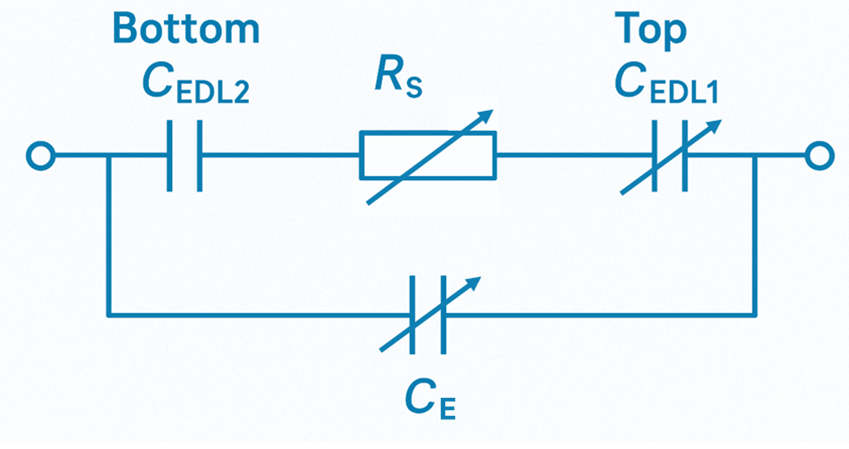


**Figure S3.** Equivalent circuit of the EDL mechanism.

The super-capacitive pressure sensor can be viewed as two EDL capacitors in series, located at the top and bottom interface between electrodes and electrolyte layer, coupling with a parallel plate capacitance. The contact resistance is denoted as Rs here.

**Figure S4**

**
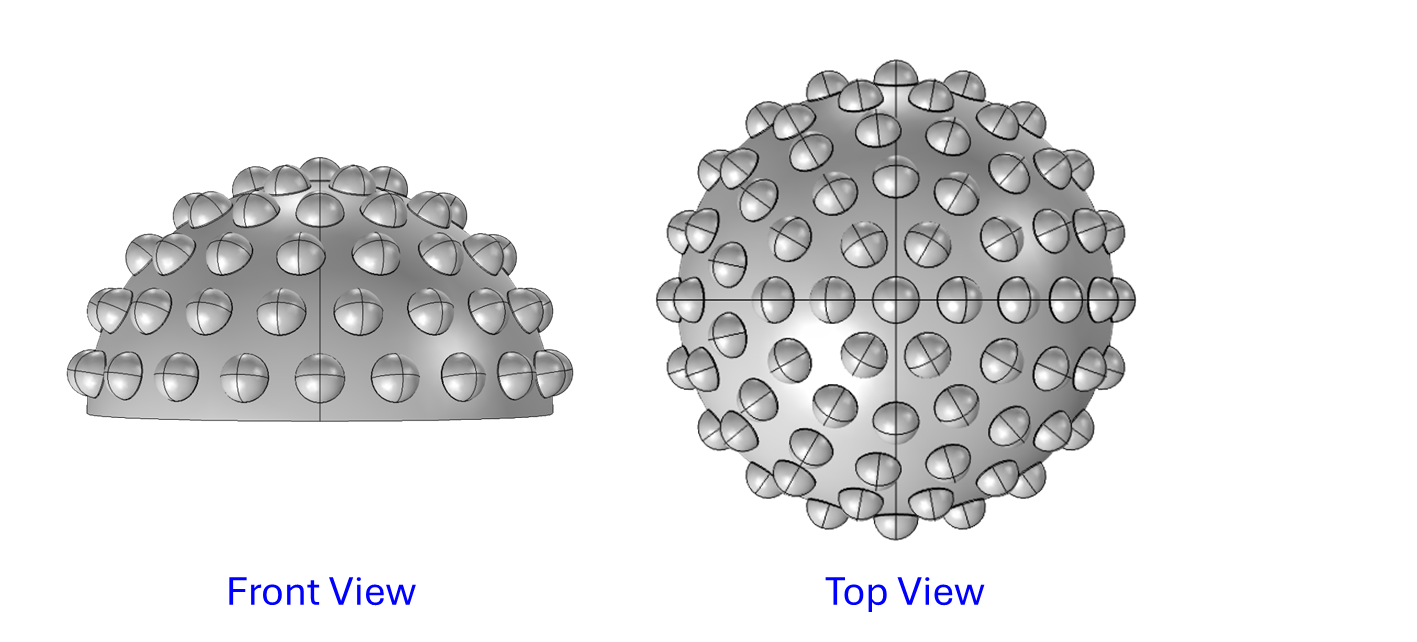
**

**Figure S4.** Three-dimensional front view and top view of the single hierarchical hemisphere-based structure.

The front view and top view of a single hierarchical structure show that the small hemispheres are regularly distributed on the surface of a large hemisphere.

**Figure S5**


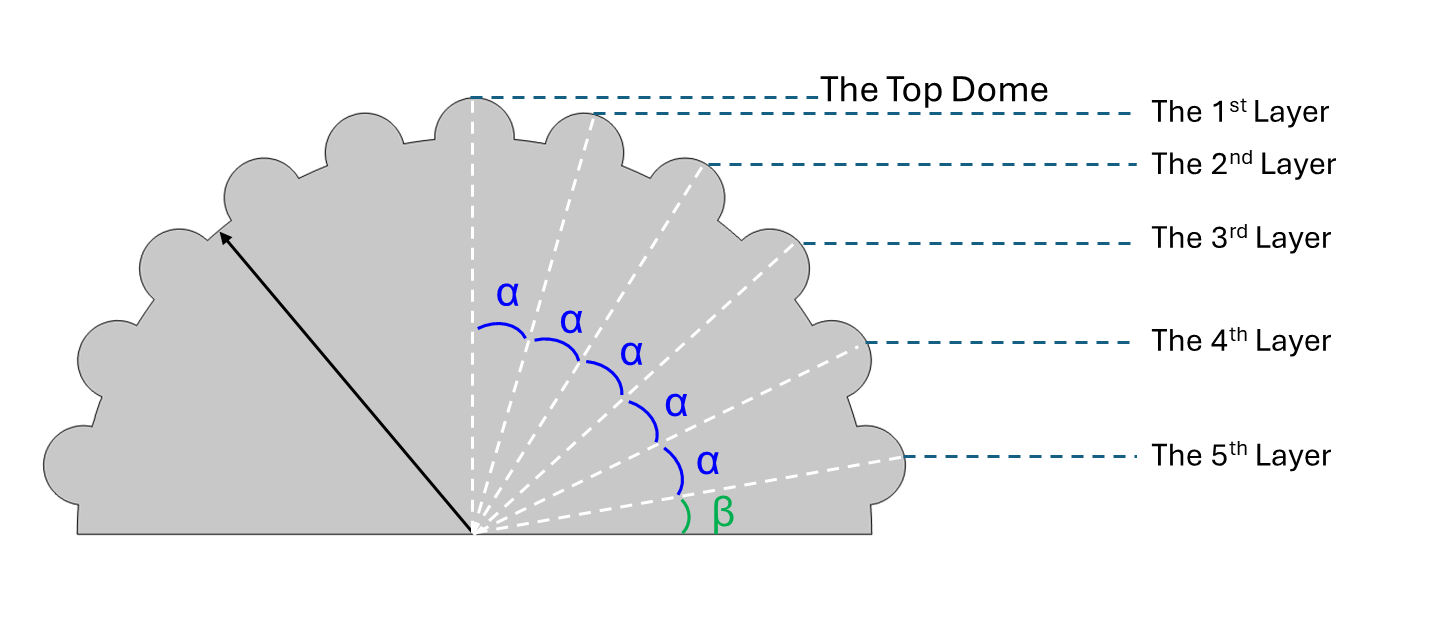


**Figure S5.** Demonstration of layers for hierarchical hemisphere structure.

According to Figure S3, there are five layers of a single hierarchical hemisphere structure. The radius of each layer, R_L_, can be expressed as Eq. S1

$$R_{L}=\sin\left( n\alpha\right)\cdot R S1$$

where n and R denote the number of the layer and radius of the large hemisphere. Then the perimeter of each layer, L, can be calculated via Eq. S2

$$L=2\pi\cdot\sin\left( n\alpha\right)\cdot R S2$$

Finally, the number of small hemispheres in each layer can be summarized as Eq. S3

$$N\approx\frac{2\pi\cdot\sin\left( n\alpha\right)\cdot R}{2r+d} S3$$

**Figure S6**


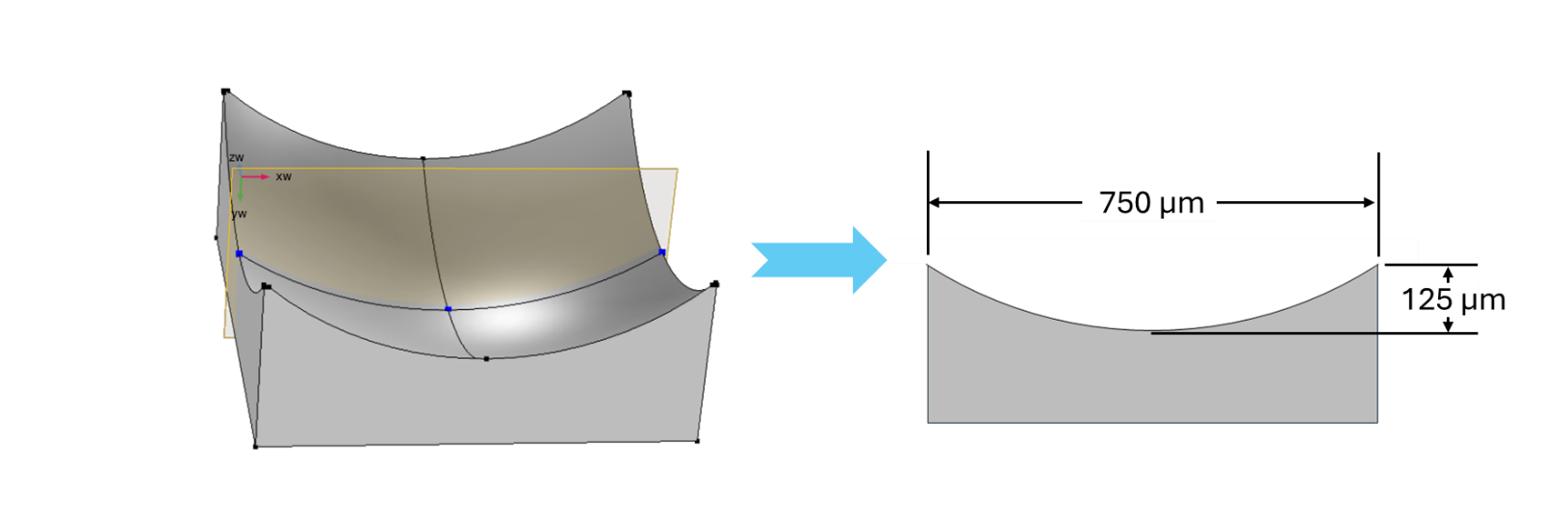


**Figure S6.** Geometry of single unit cell of curvy top electrode for super-capacitive pressure sensor.

**Figure S7**


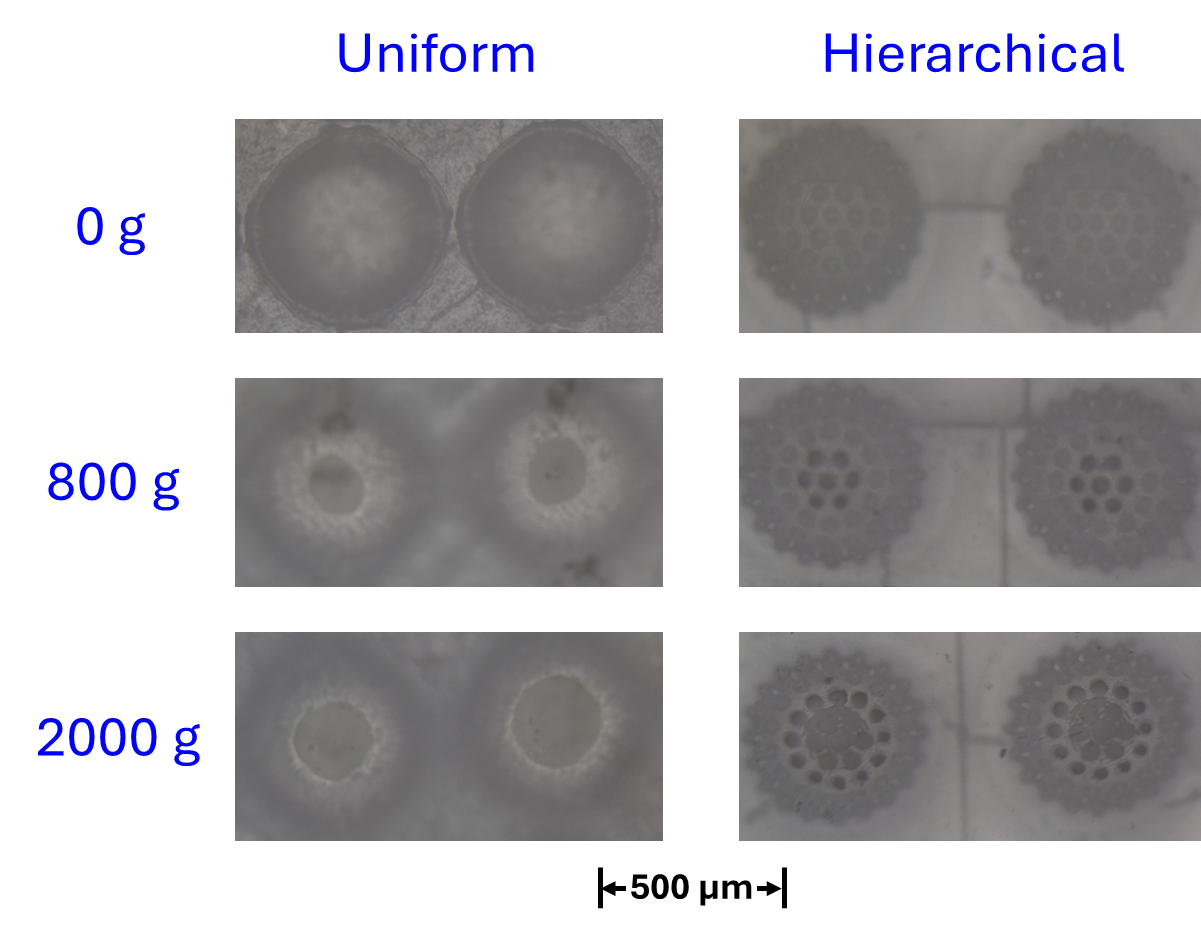


**Figure S7.** Contact area variation of both hierarchical and non-hierarchical hemispheres under compression via optical microscopy observation.

**Figure S8**


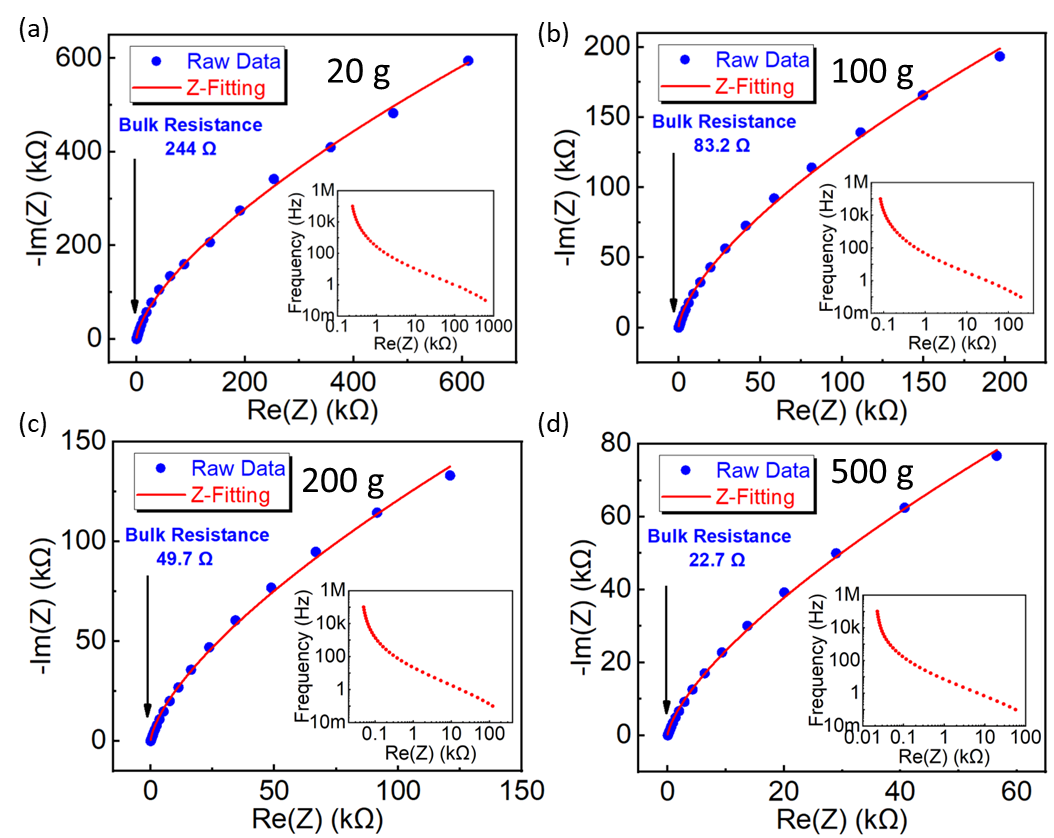


**Figure S8.** Nyquist plots for electrolyte layer with non-hierarchical hemispheres under weights of (a) 20 g (b) 100 g (c) 200 g and (d) 500 g.

**Figure S9**

**
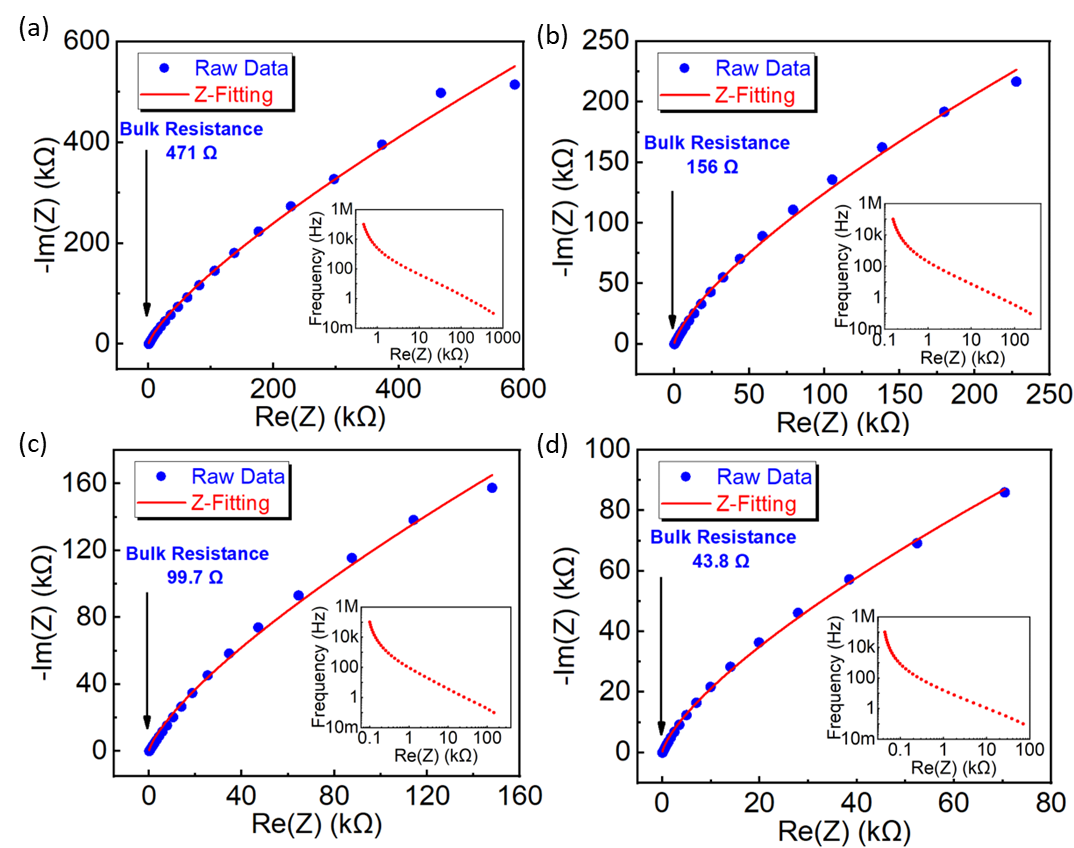
**

**Figure S9.** Nyquist plots for electrolyte layer with hierarchical hemispheres under weights of (a) 20 g (b) 100 g (c) 200 g and (d) 500 g.

**Figure S10**


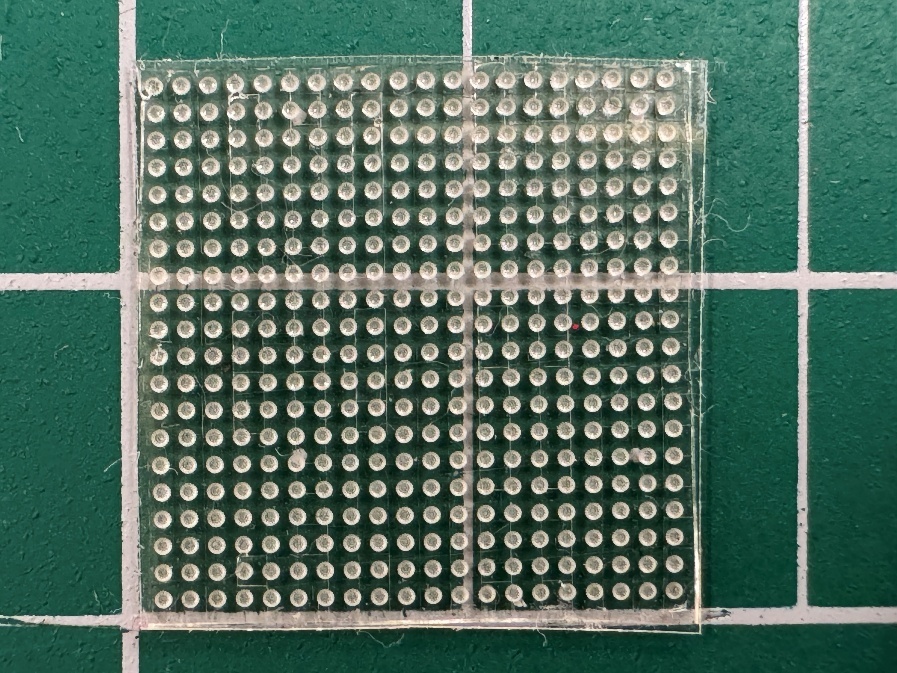


**Figure S10.** Electrolyte layer with hierarchical hemisphere structure in 20×20 arrays.

**Figure S11**


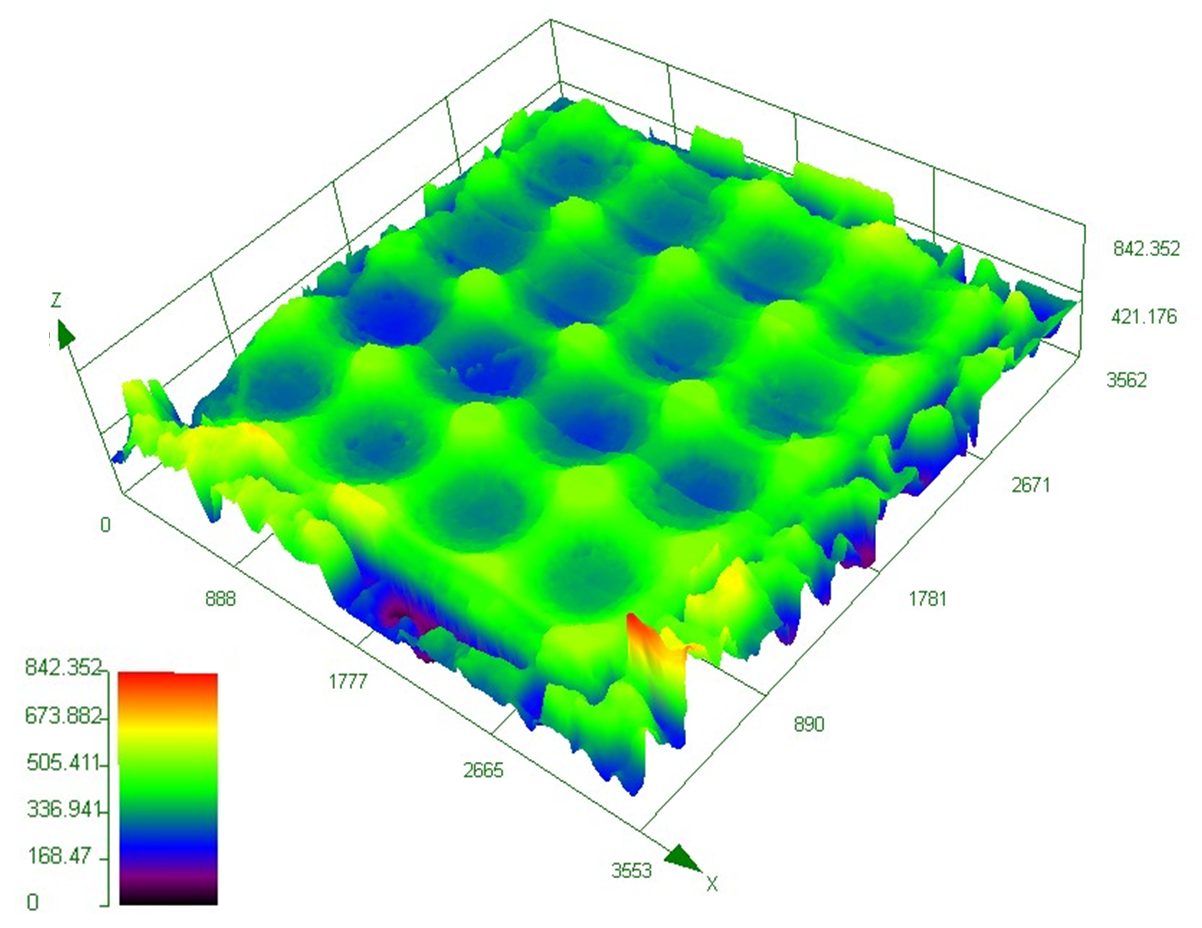


**Figure S11.** Scanning profile for curvy surface of top electrode used for super-capacitive pressure sensor.

**Figure S12**


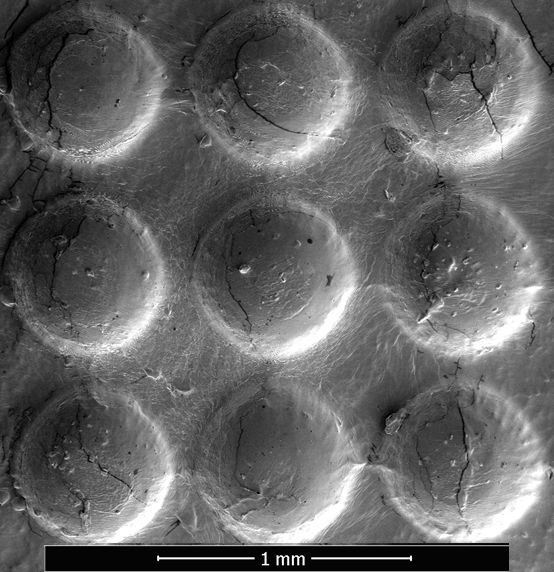


**Figure S12.** SEM characterization of uniform non-hierarchical hemispheres

**Figure S13**


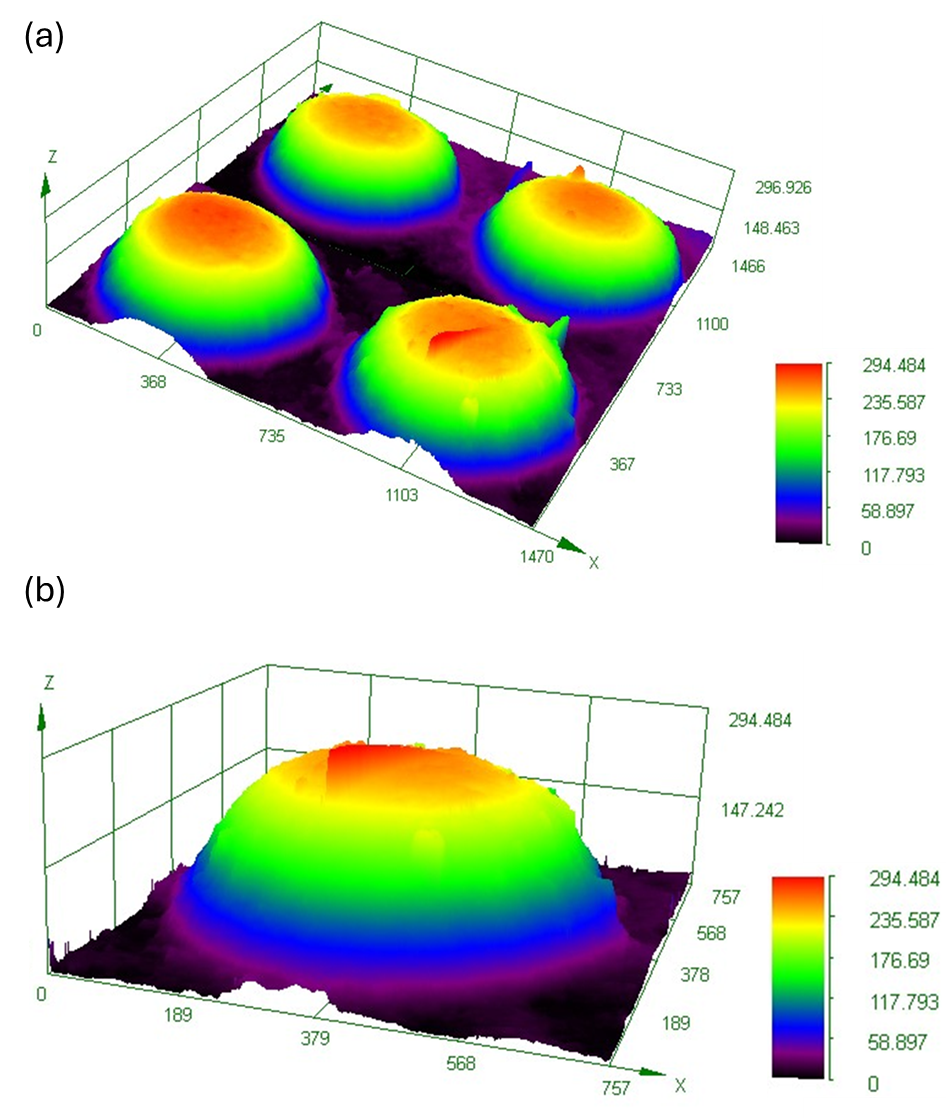


**Figure S13.** Scanning profile for uniform non-hierarchical hemisphere structure (a) 2×2 array and (b) single unit.

**Figure S14**


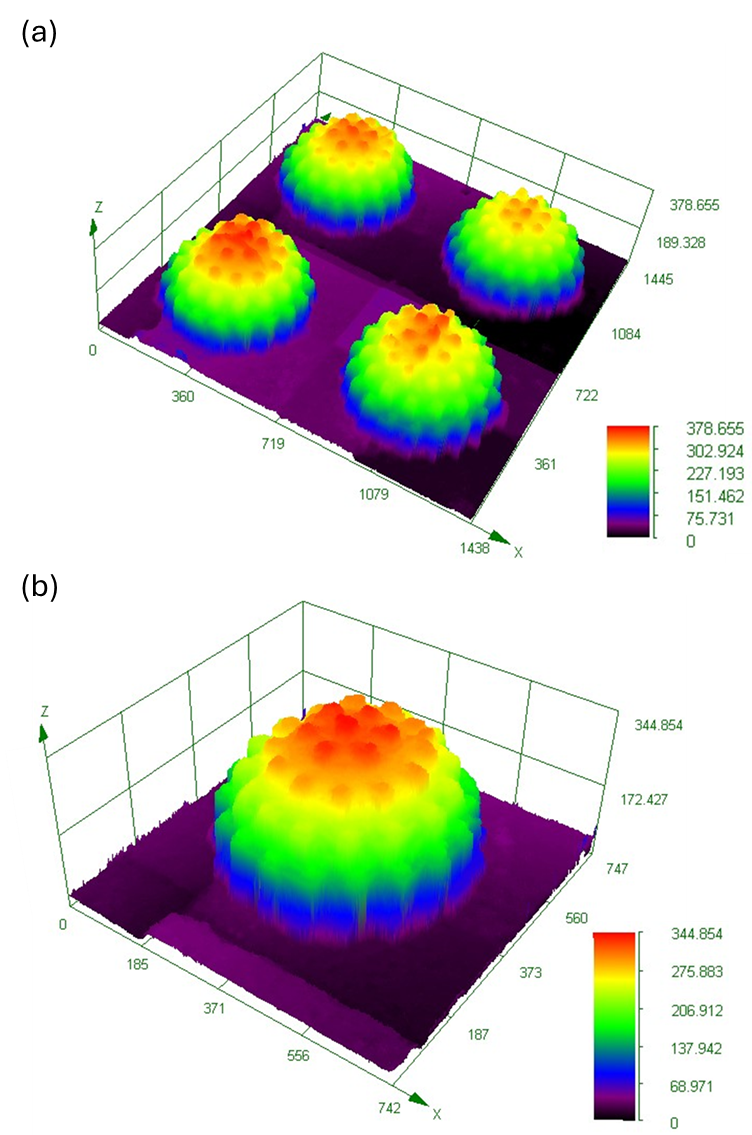


**Figure S14.** Scanning profile for hierarchical hemisphere structure (a) 2×2 array and (b) single unit.

**Figure S15**


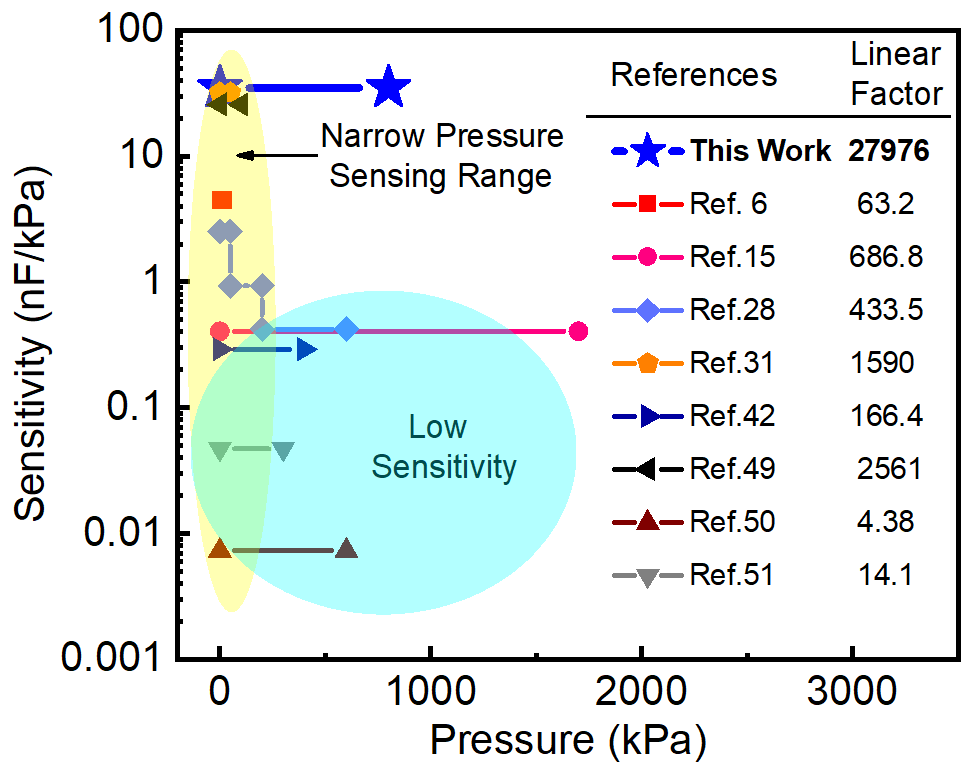


**Figure S15.** Comparison of sensitivity and linear sensing range between this work and other works.

The linear factor was defined by sensitivity, multiplied by linear sensing range, L=kꞏP, where L, k and P denote linear factor, sensitivity and linear sensing range, respectively.^[1, 2]^

**Figure S16**


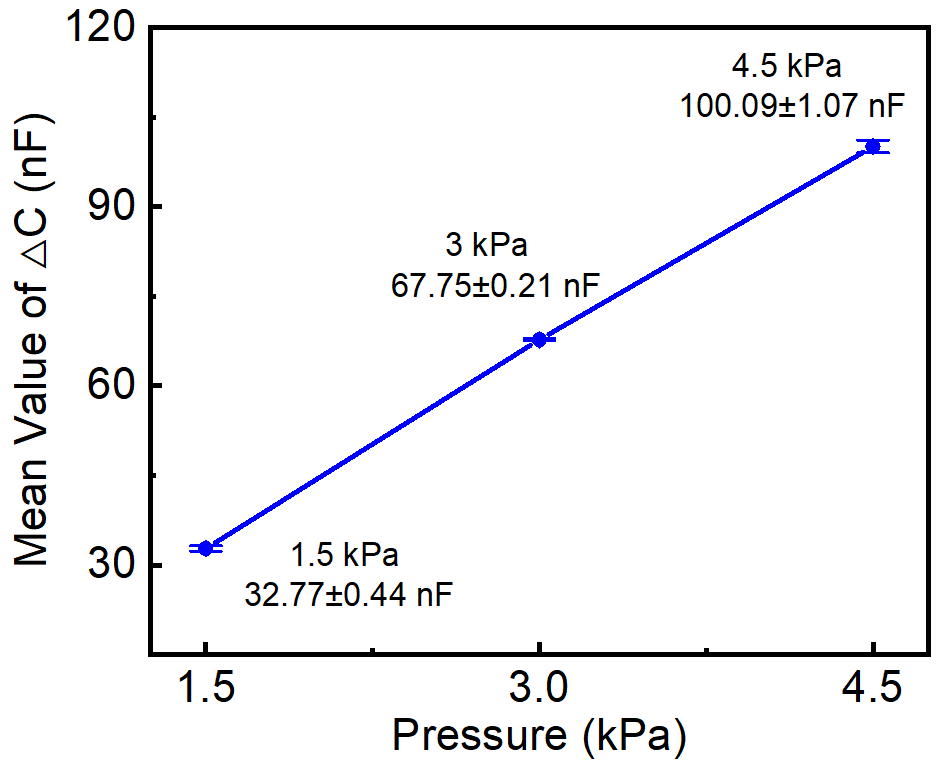


**Figure S16.** Average values of capacitance variation of super-capacitive pressure sensor under pressures of 1.5, 3 and 4.5 kPa.

**Figure S17**


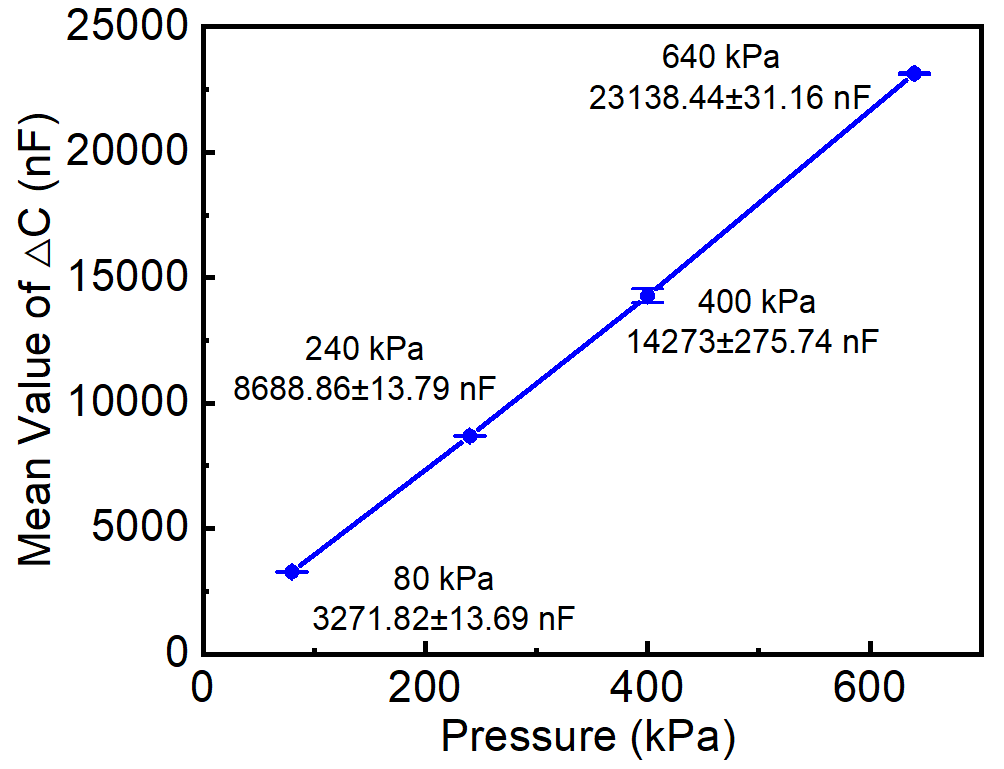


**Figure S17.** Average values of capacitance variation of super-capacitive pressure sensor under pressures of 80, 240, 400 and 640 kPa.

**Figure S18**


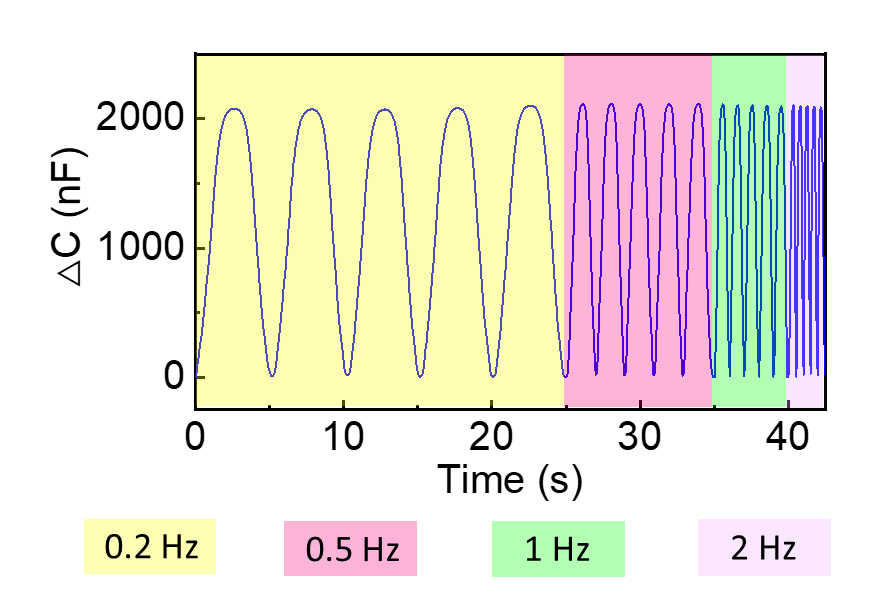


**Figure S18.** Capacitance signal output of super-capacitive pressure sensor under pressure of 60 kPa with mechanical frequencies from 0.2 Hz to 2 Hz.

**Figure S19**


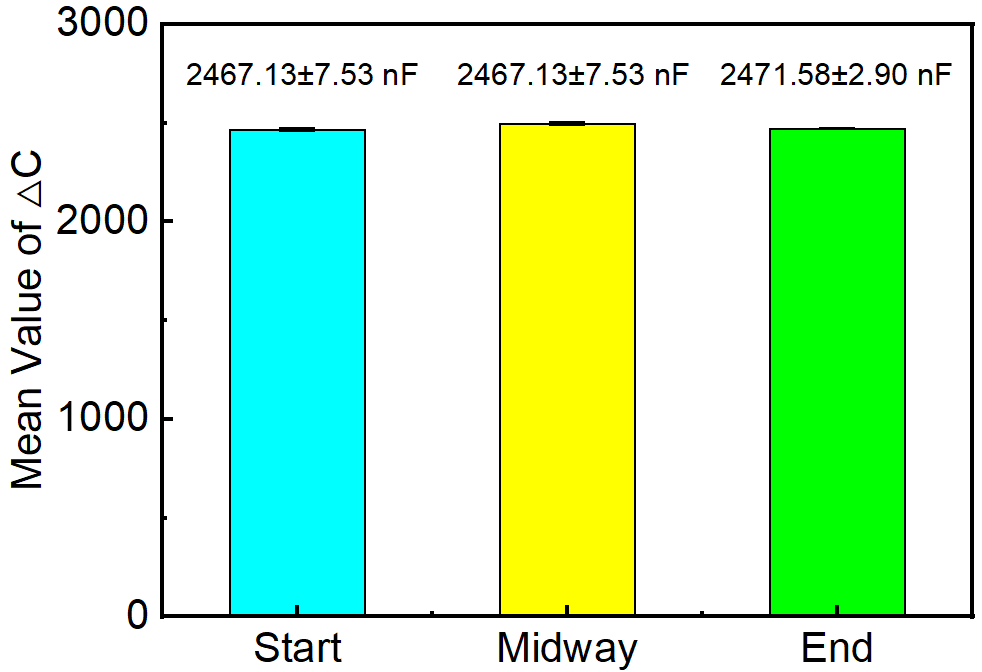


**Figure S19.** Mean values of capacitance variation for ten-cycles at start, midway and end of 5000-cycle cyclic loading, under pressure of 60 kPa.

**Figure S20**


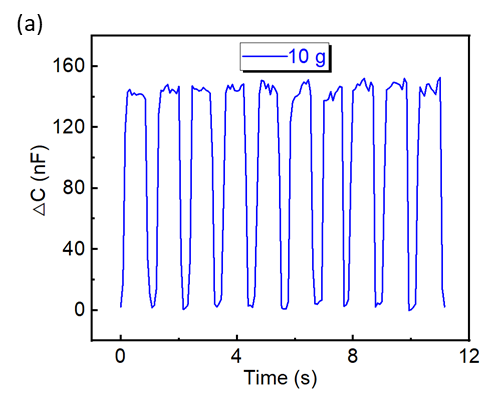


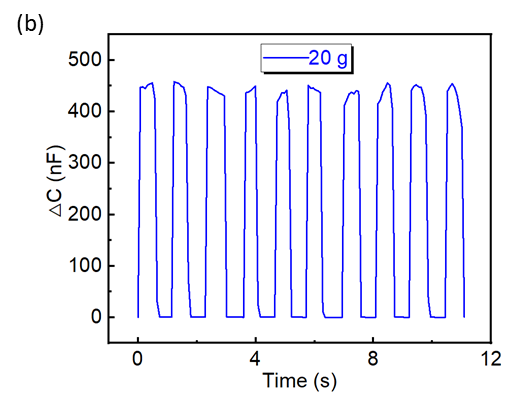


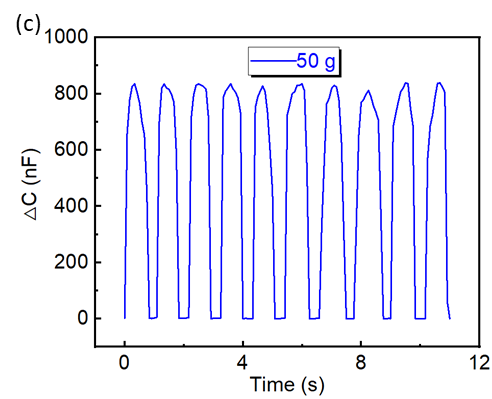


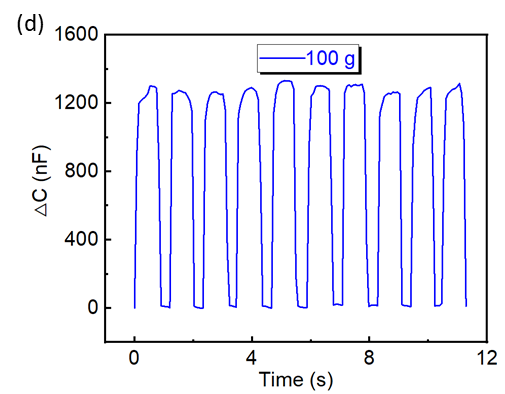


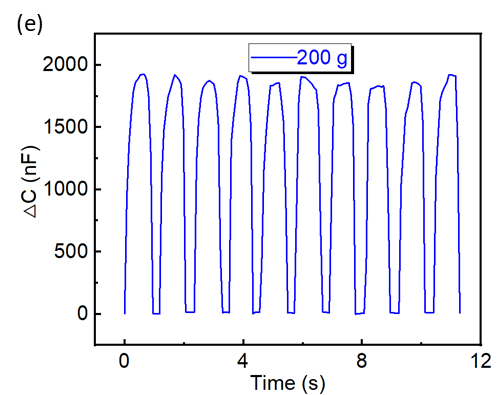


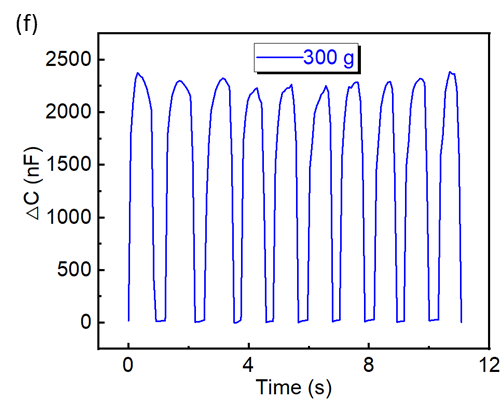


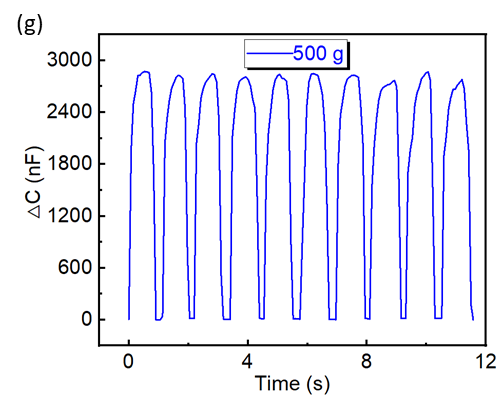


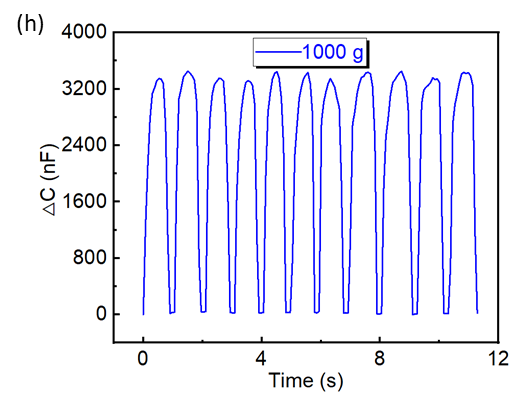


**Figure S20.** Capacitance signal output for grasping objects with different weights (a) 10 g (b) 20 g (c) 50 g (d) 100 g (e) 200 g (f) 300 g (g) 500 g and (h) 1000 g.

**Figure S21**

**
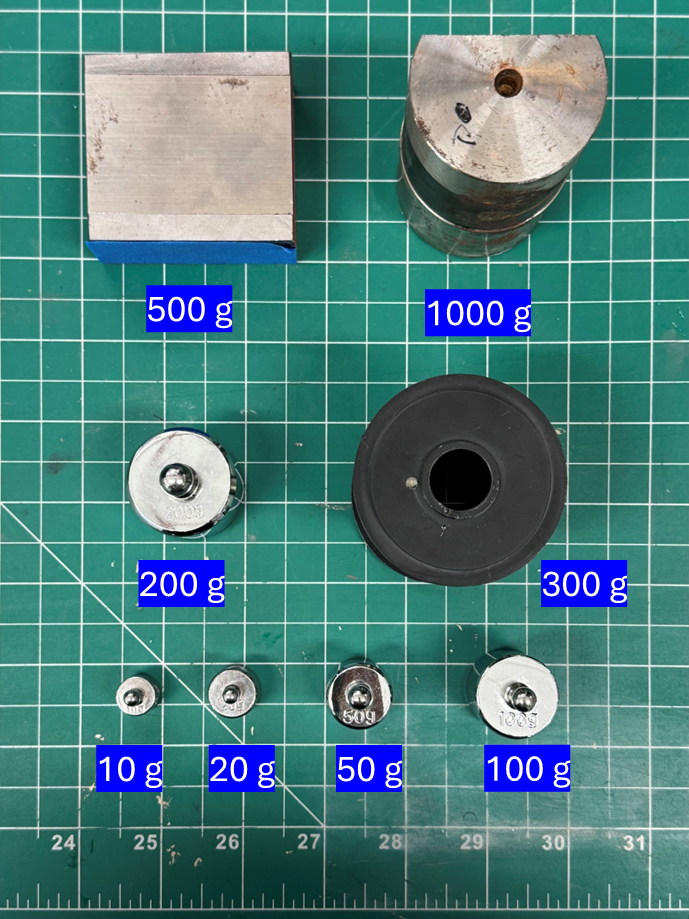
**

**Figure S21.** Objects with different weights (a) 10 g (b) 20 g (c) 50 g (d) 100 g (e) 200 g (f) 300 g (g) 500 g and (h) 1000 g.

**Figure S22**

**
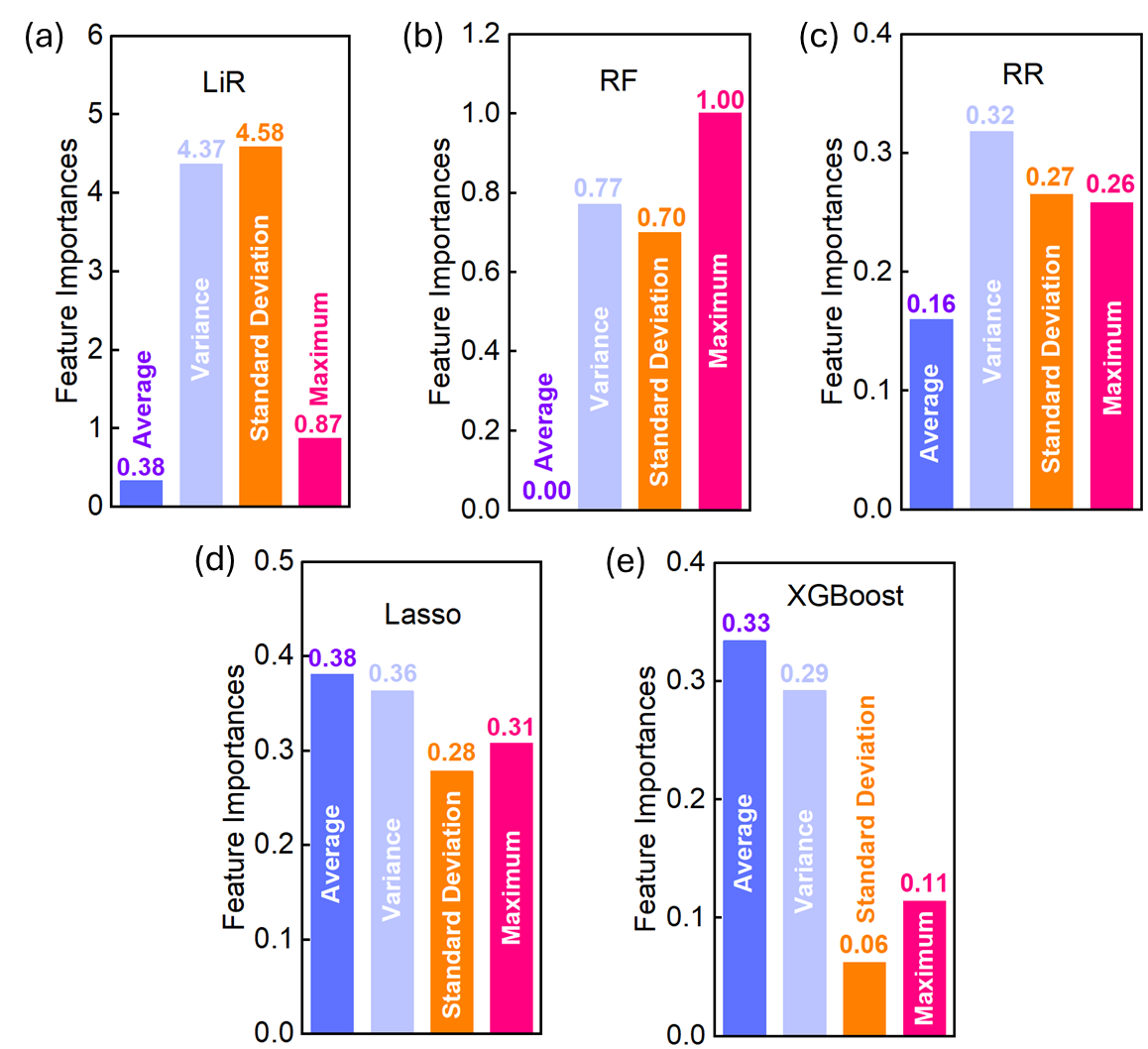
**

**Figure S22.** Feature importances scores of regression models of (a) LiR (b) RF (c) RR (d) Lasso (e) XGBoost, for weight prediction of various objects.

**Figure S23**

**
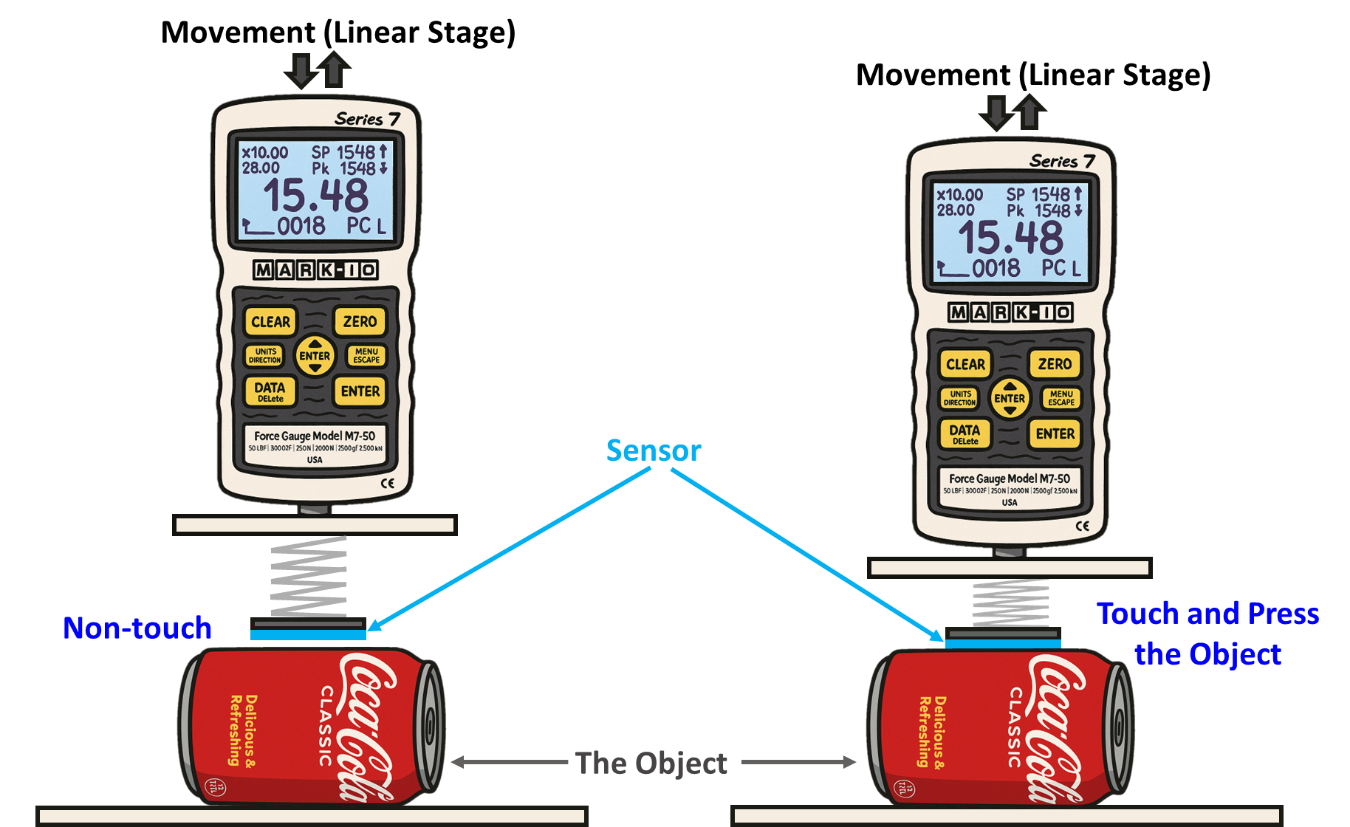
**

**Figure S23.** Schematic demonstration of super-capacitive sensor touches and pressed the object.

**Figure S24**

**
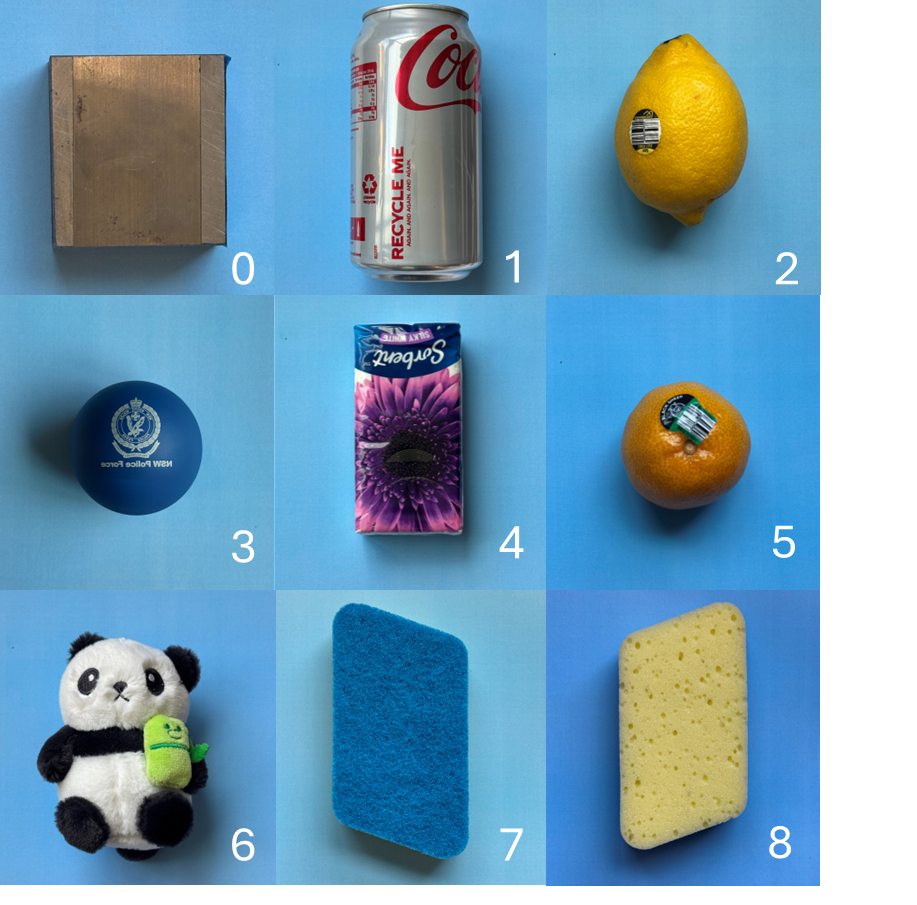
**

**Figure S24.** Nine different objects for recognition by super-capacitive pressure sensor using machine learning.

**Figure S25**

**
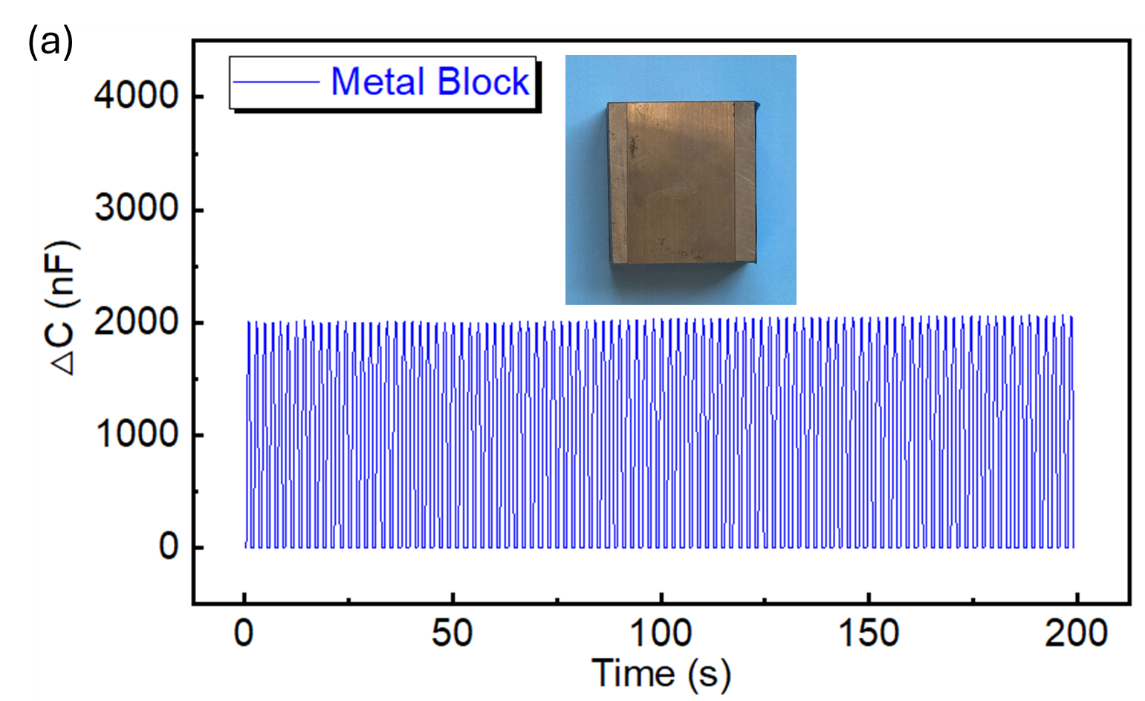
**

**
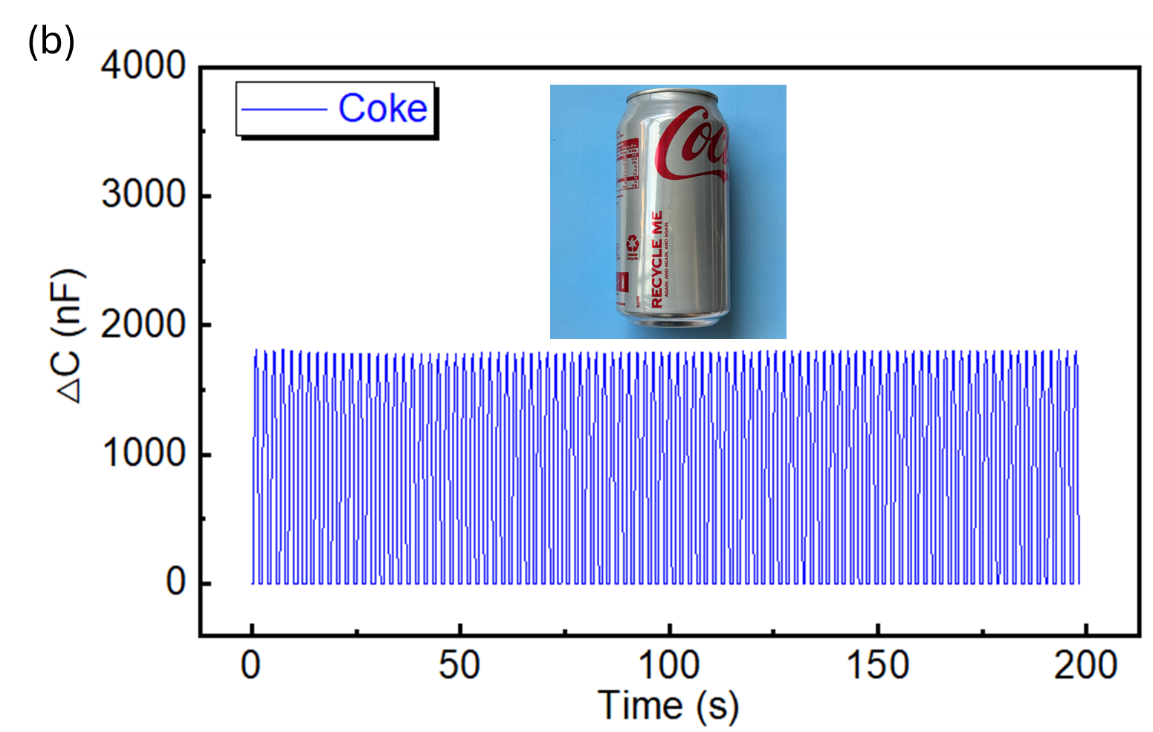
**

**
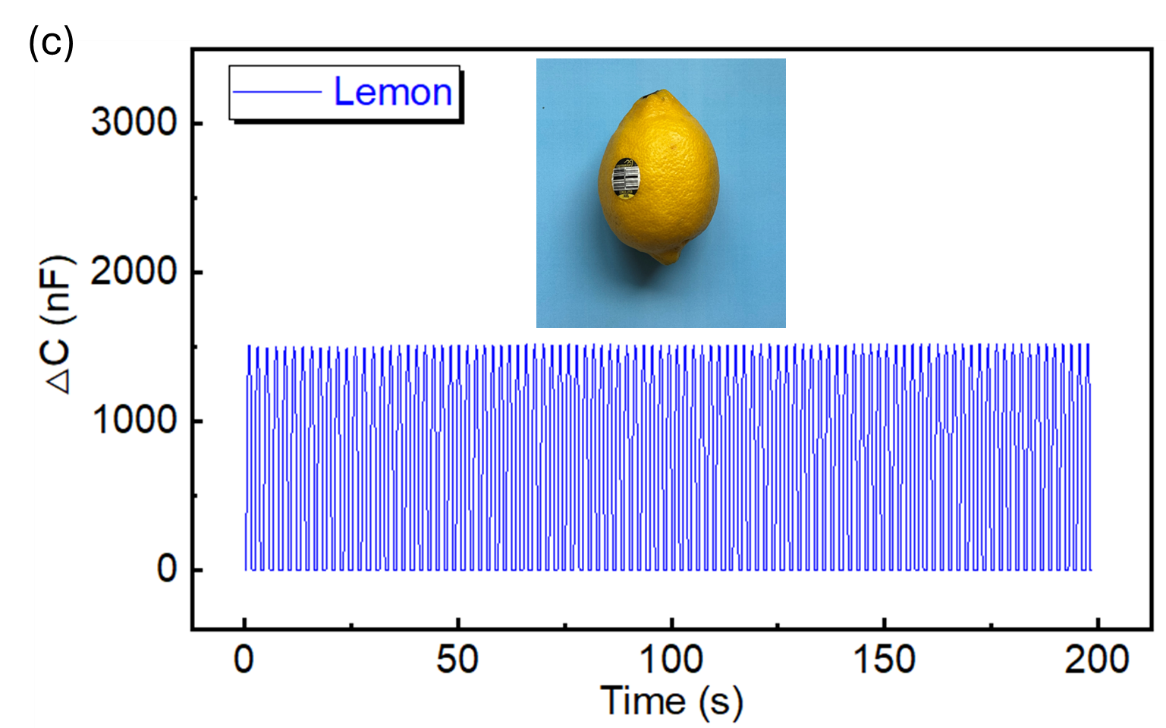
**

**
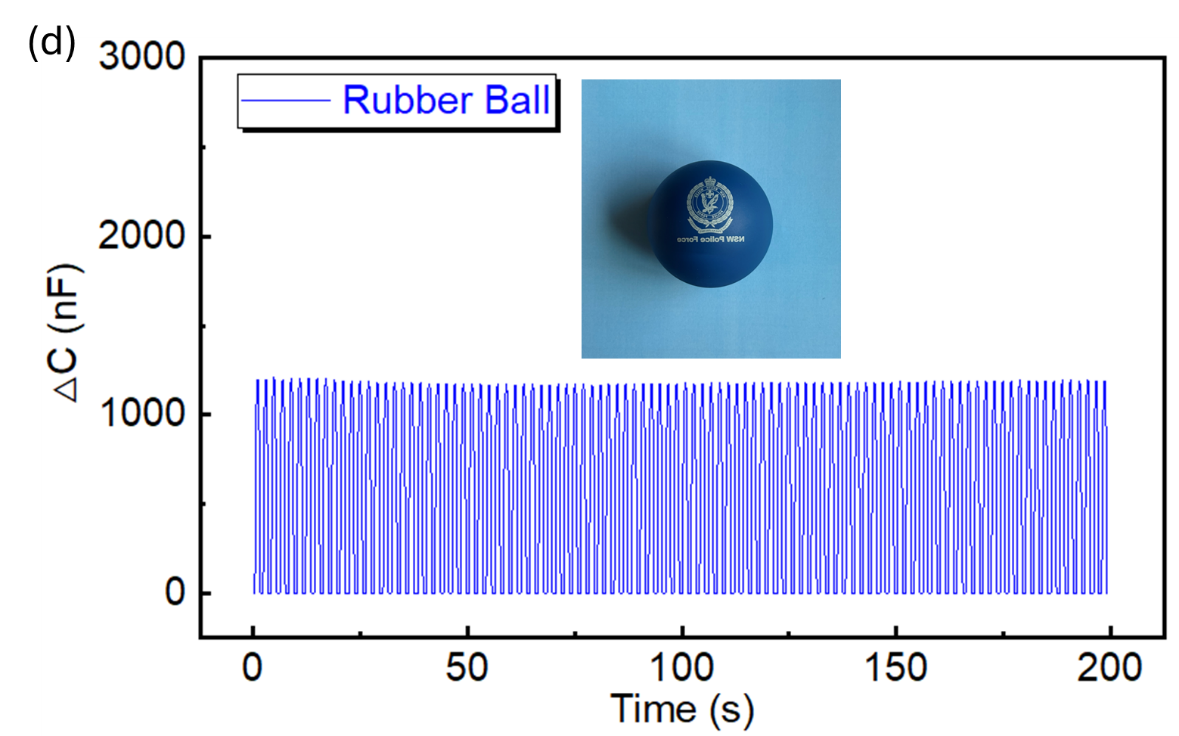
**

**
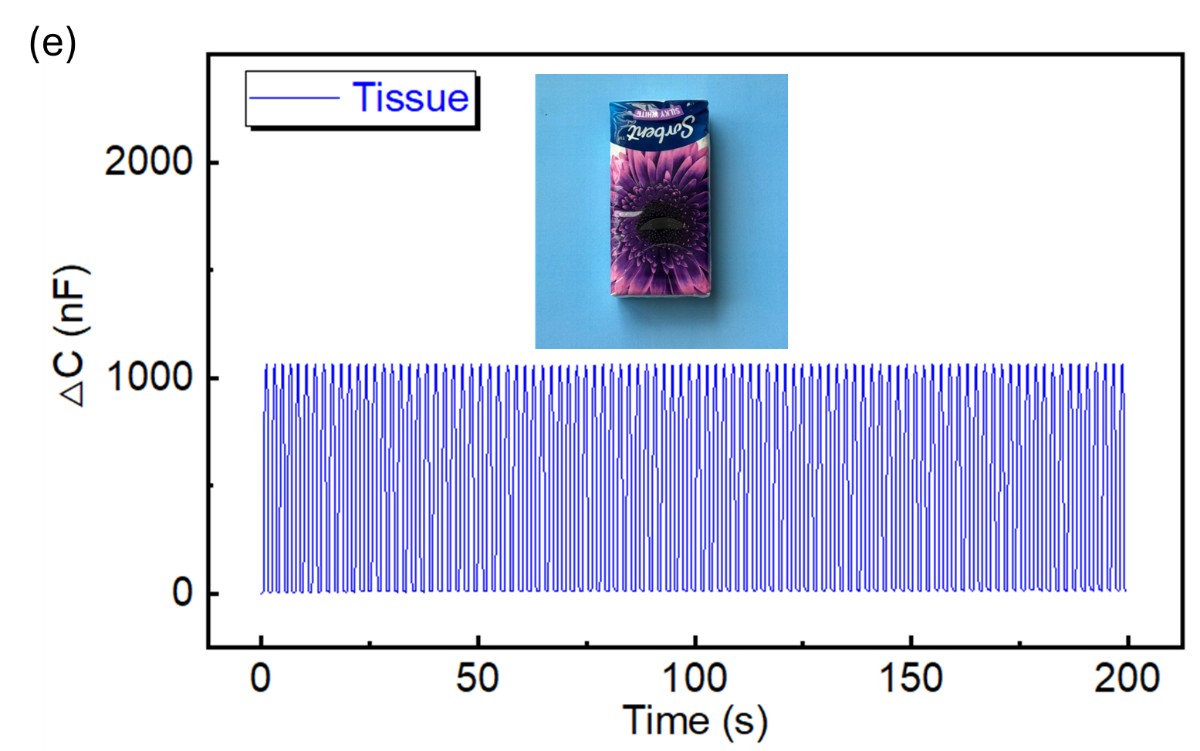
**

**
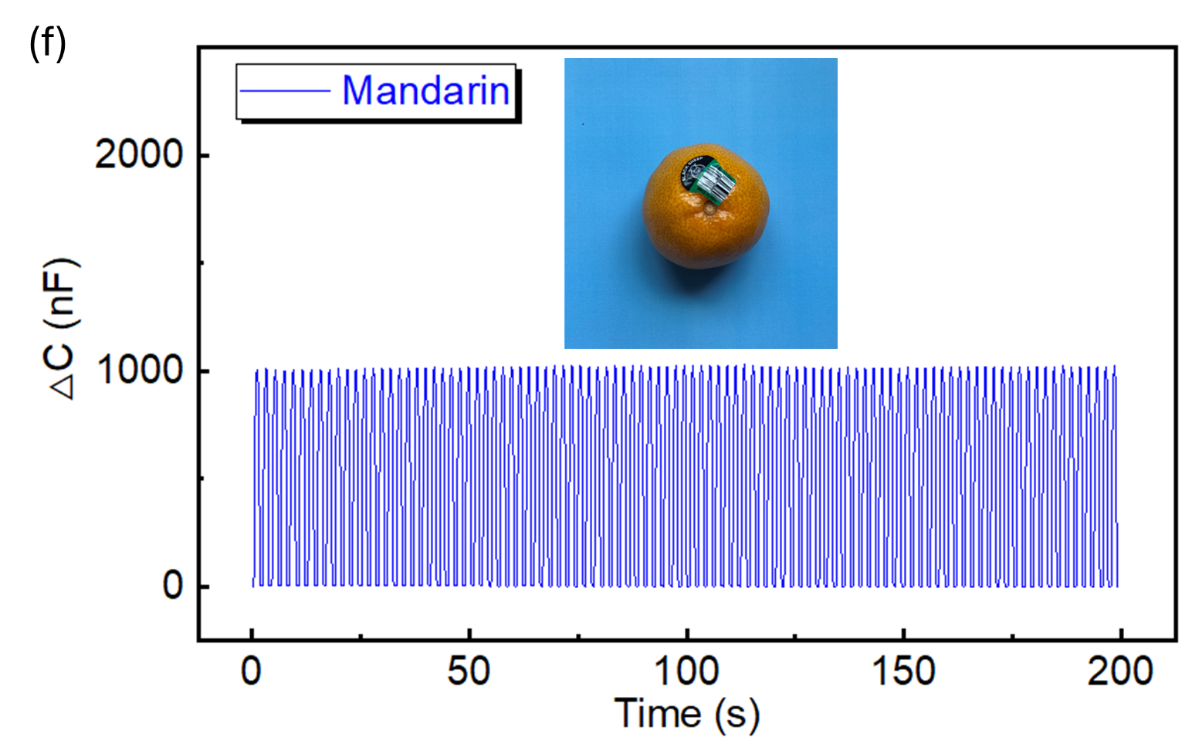
**

**
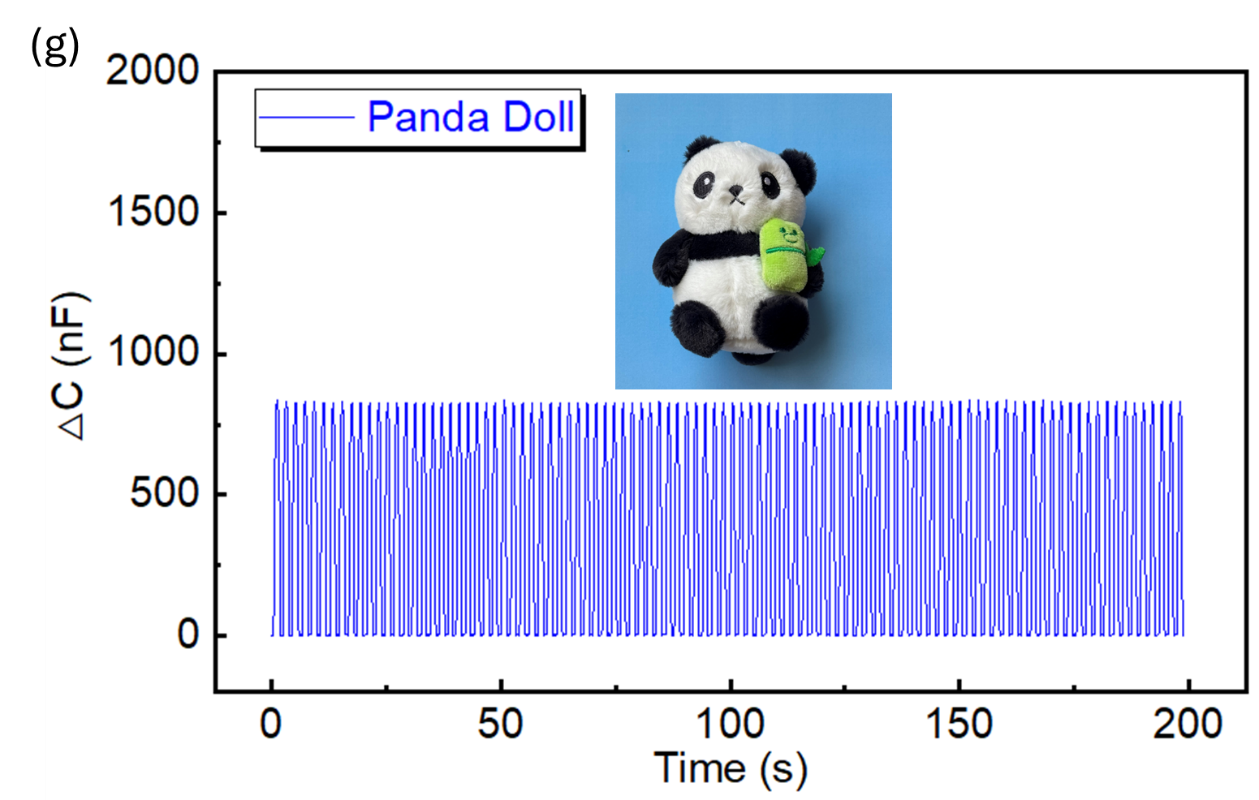
**

**
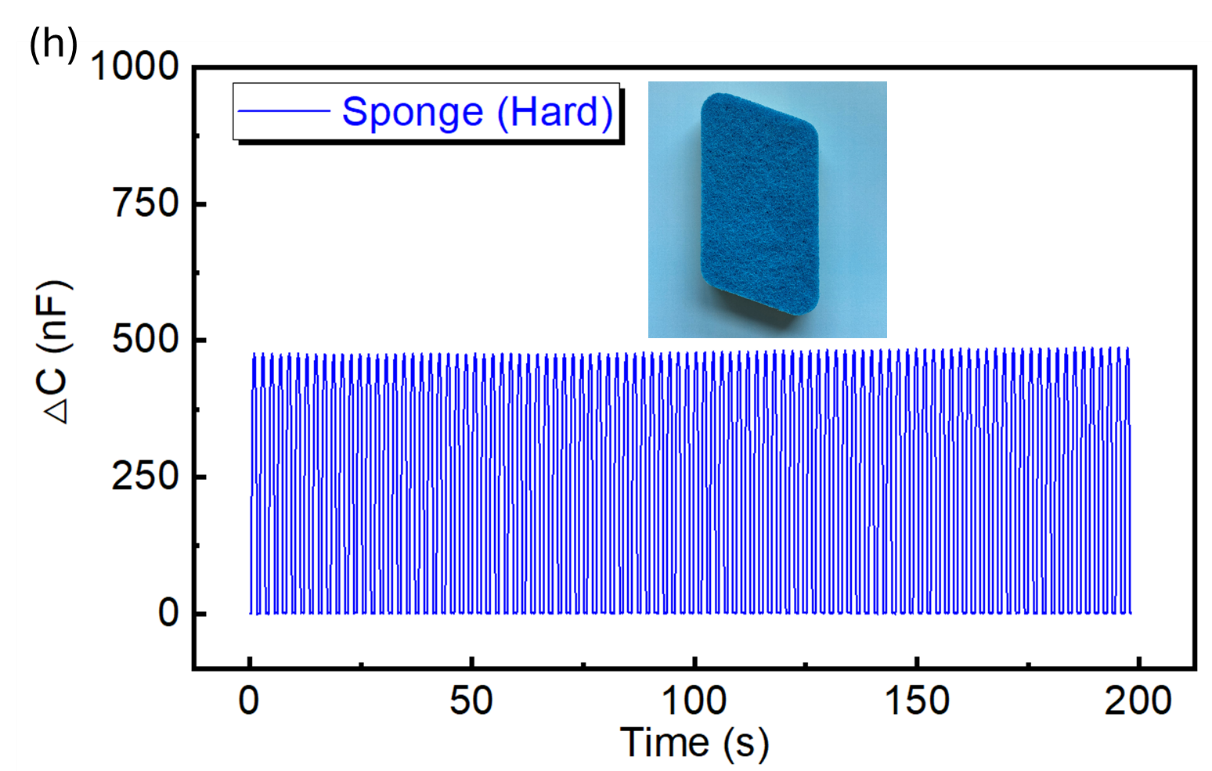
**

**
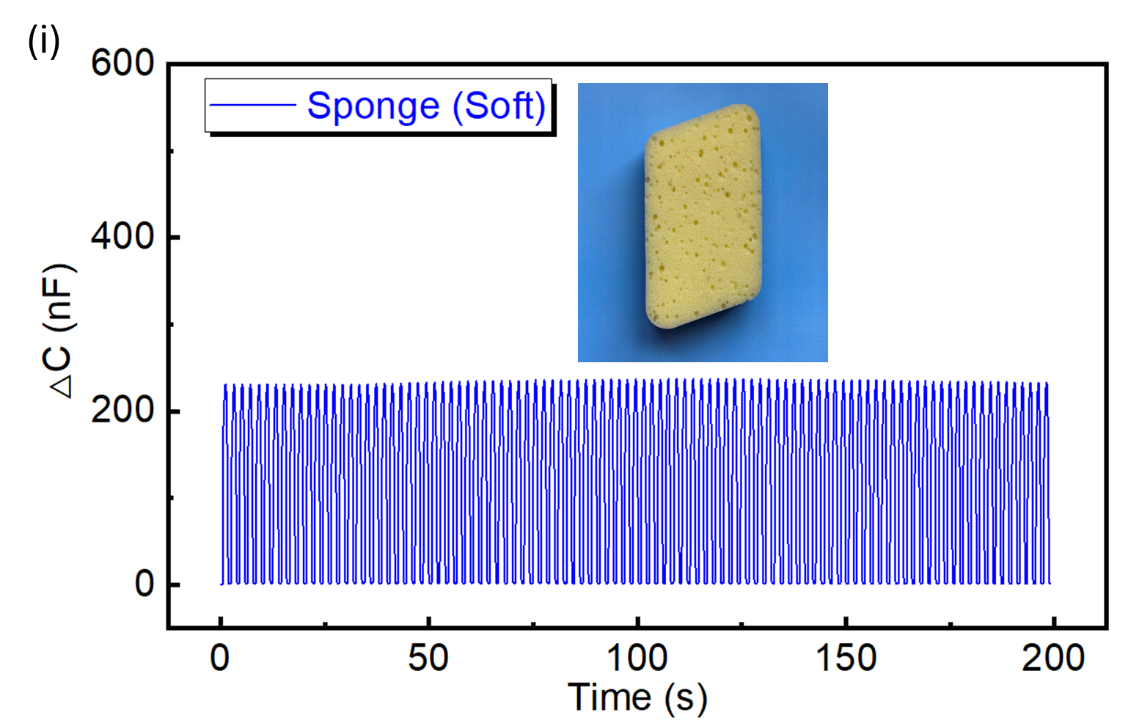
**

**Figure S25.** Capacitance response of super-capacitive pressure sensor when touching and pressing objects of (a) metal block (b) coke (c) lemon (d) rubber ball (e) tissue (f) mandarin (g) panda doll (f) hard sponge (g) soft sponge.

**Figure S26**

**
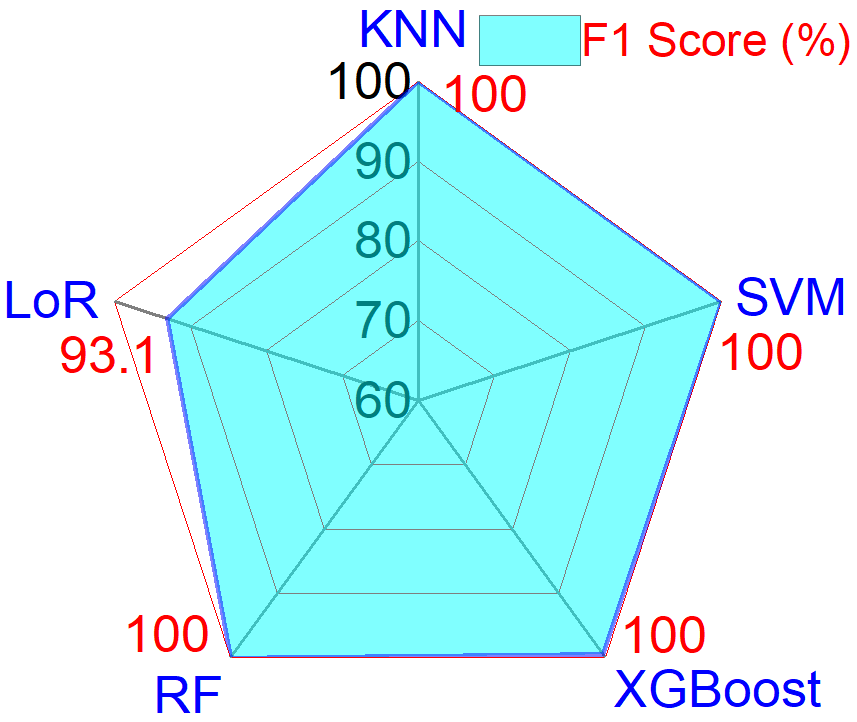
**

**Figure S26.** F1 score for classification models of KNN, SVM, XGBoost, RF and LoR.

**Figure S27**

**
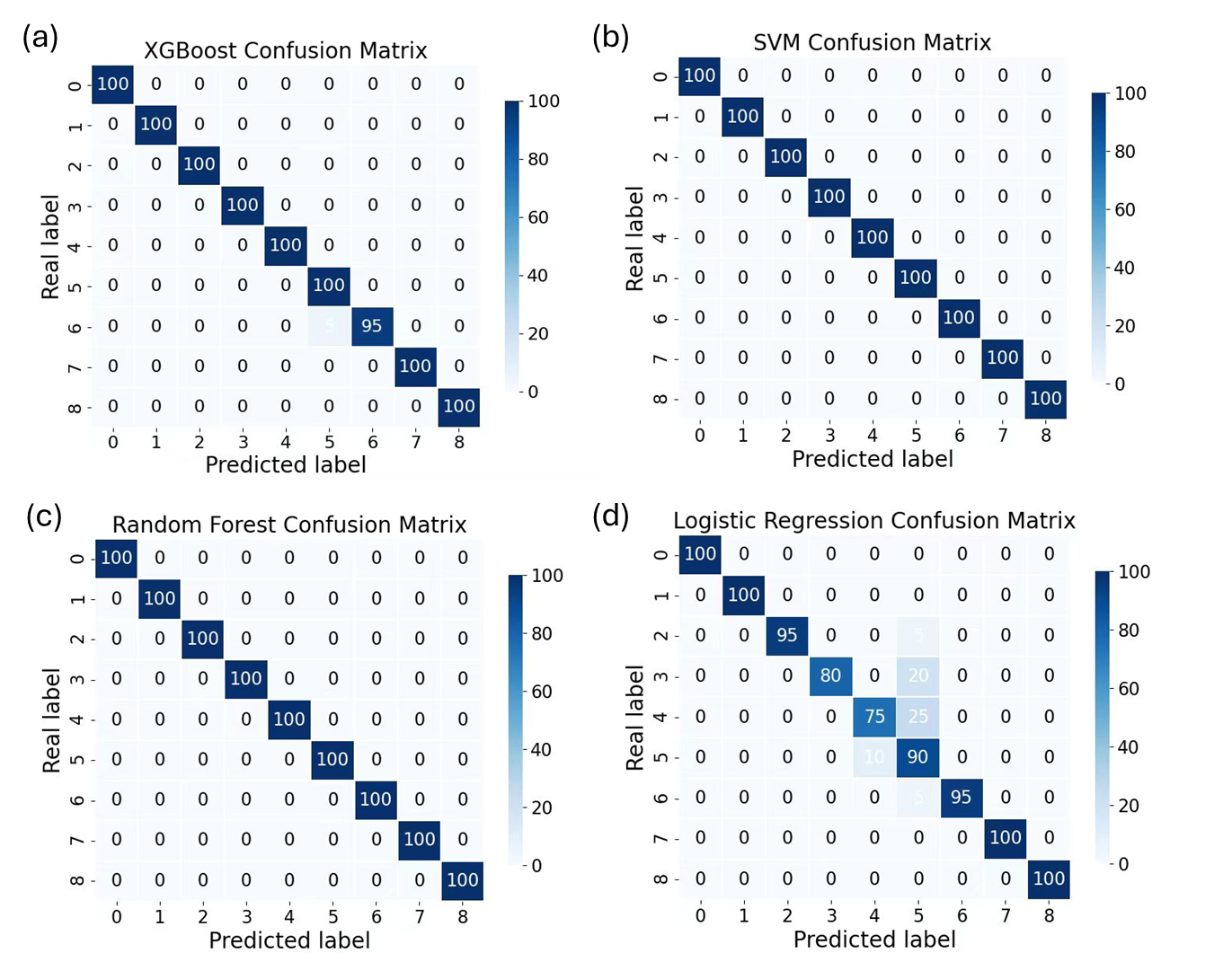
**

**Figure S27.** Confusion matrix for classification models of (a) XGBoost (b) SVM) (c) RF and (d) LR, for objects recognition, with 100 sets for each category of the object.

**Figure S28**

**
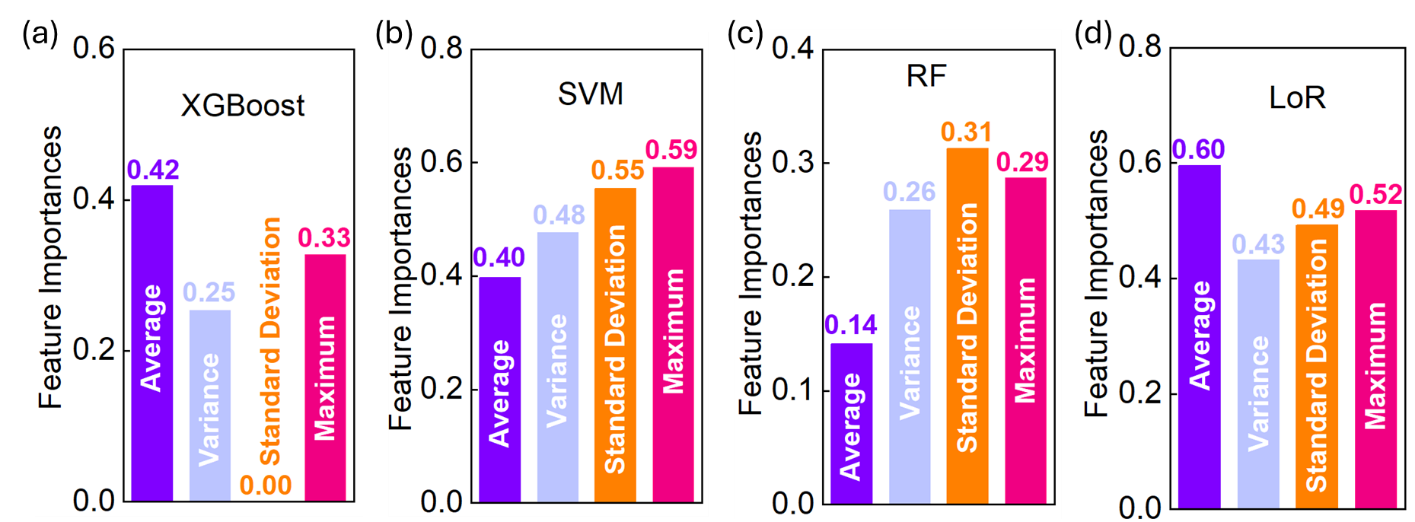
**

**Figure S28.** Feature importances for classification models of (a) XGBoost (b) SVM) (c) RF and (d) LR, for object recognition, with 100 sets for each category of the object.

**Figure S29**


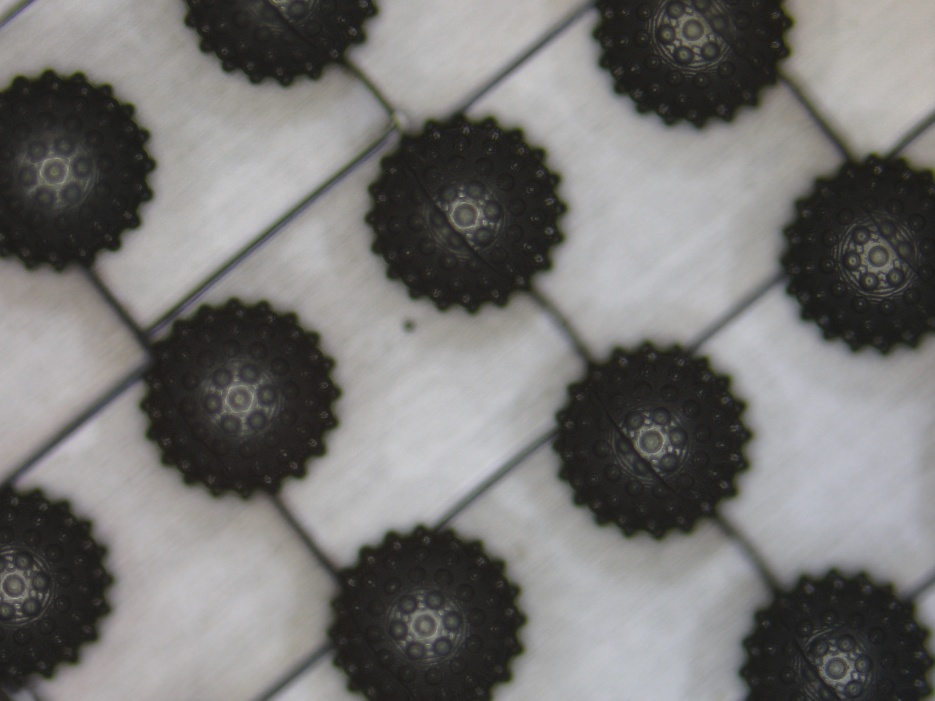


**Figure S29.** Hierarchical hemisphere structure 3D printed by 3D laser lithography system (Nanoscribe).

**Figure S30**


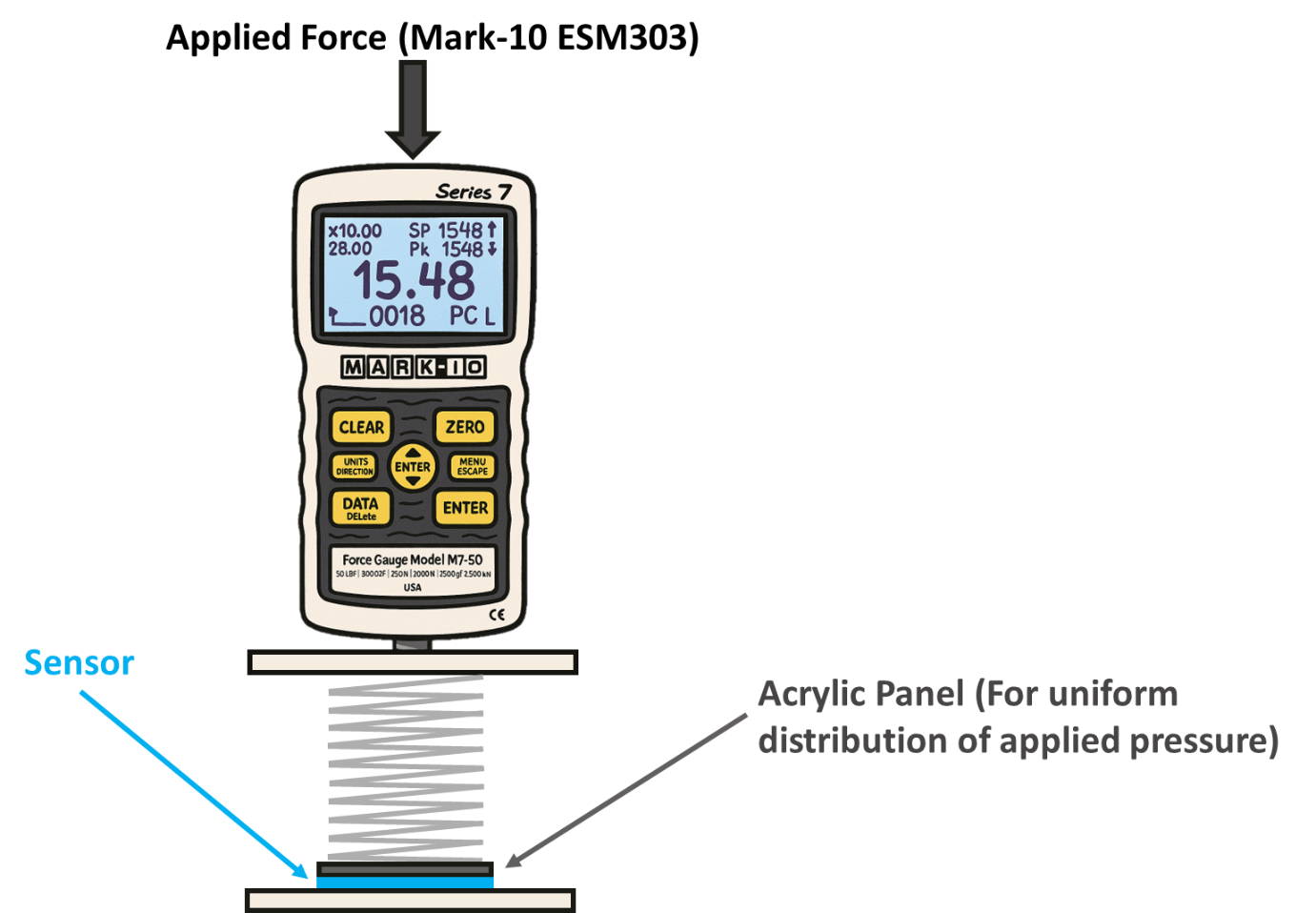


**Figure S30.** Schematic illustration of pressure supply sensitivity characterization of super-capacitive pressure sensors.

**Figure S31**


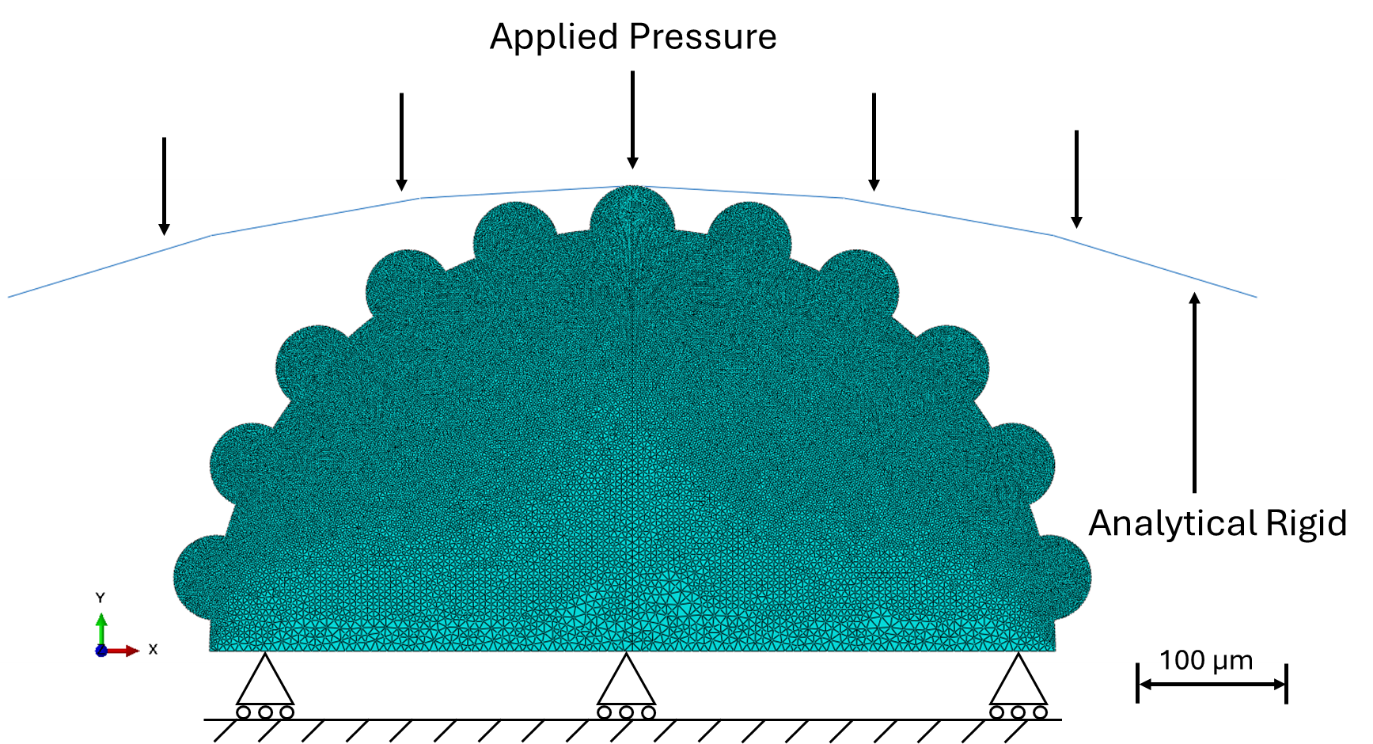


**Figure S31.** FE model of a hierarchical electrolyte layer in contact with a curvy-surface electrode.

**Table S1**

**Table S1.** Comparison of Sensing Performance among Different Works


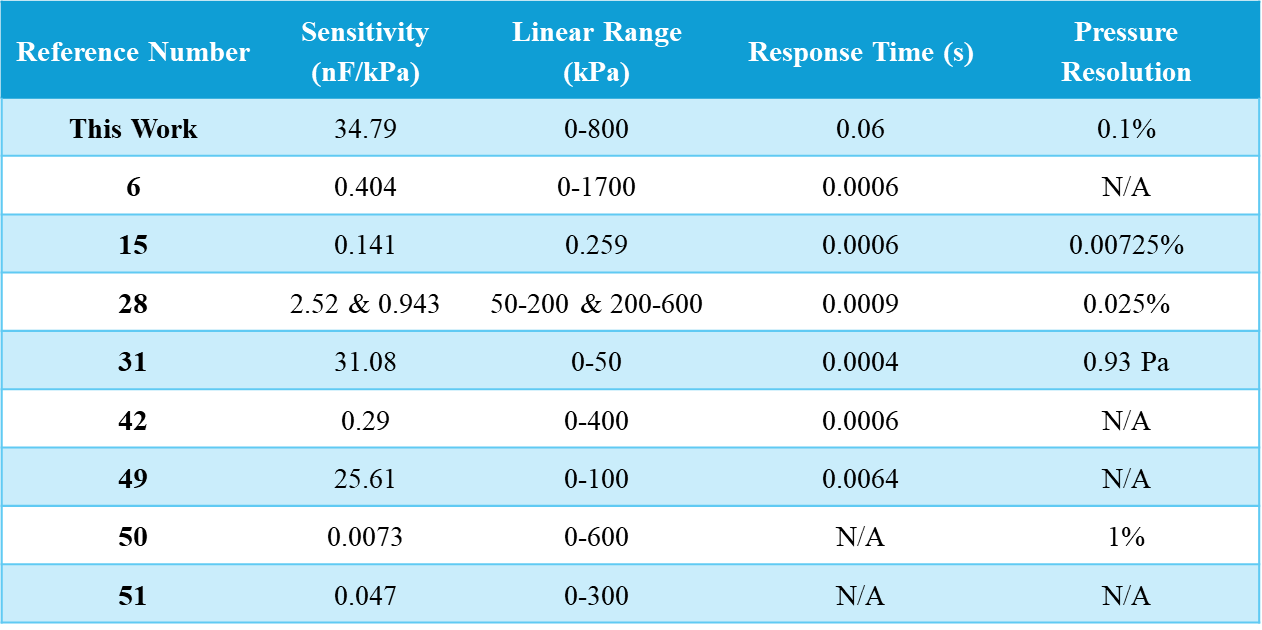


**Table S2**

**Table S2.** Feature Importance Score for Various Regression Models

| Feature Importance Score | Average | Variance | Standard Deviation | Maximum |
| --- | --- | --- | --- | --- |
| KNN | 0.381 | 0.363 | 0.308 | 0.278 |
| LiR | 0.333 | 4.371 | 4.582 | 0.872 |
| RF | 0 | 0.77 | 0.7 | 1 |
| RR | 0.16 | 0.318 | 0.265 | 0.258 |
| Lasso | 0.381 | 0.363 | 0.278 | 0.308 |
| XGBoost | 0.334 | 0.292 | 0.062 | 0.114 |

**Table S3**

**Table S3.** Feature Importance Score for Various Classification Models

| Feature Importance Score | Average | Variance | Standard Deviation | Maximum |
| --- | --- | --- | --- | --- |
| KNN | 0.478 | 0.483 | 0.548 | 0.594 |
| LoR | 0.595 | 0.432 | 0.492 | 0.518 |
| RF | 0.141 | 0.259 | 0.313 | 0.287 |
| SVM | 0.397 | 0.476 | 0.554 | 0.591 |
| XGBoost | 0.419 | 0.253 | 0 | 0.327 |

**Note S1**

The regression models in machine learning are generally evaluated by three indexes, coefficient of determination (R^2^), root-mean-square error (RMSE) and mean absolute error (MAE). The definition of R^2^, RMSE and MAE can be expressed by Eq. S4, Eq. S5 and Eq. S6, respectively.

$$R^{2}=1-\frac{\sum_{i=1}^{n} \left( y_{i}-\hat{y}_{i} \right)^{2}}{\sum_{i=1}^{n} \left( y_{i}-\bar{y} \right)^{2}} S4$$

$$RMSE=\sqrt{\sum_{i=1}^{n} \frac{{(\hat{y}_{i}-y_{i})}^{2}}{n}} S5$$

$$MAE=\frac{1}{n}\sum_{i=1}^{n} \left| y_{i}-\hat{y}_{i} \right| S6$$

where *i*, *n*, $y_{i}$, $\hat{y}_{i}$ and $\bar{y}$ denote observation, number of observations, observed values, predicted values and the mean value of observed values, respectively.

References:

[1] A. J. Cheng, W. Chang, Y. Qiao, F. Huang, Z. Sha, S. He, L. Wu, D. Chu, S. Peng, ACS Appl. Mater. Interfaces 2024, 16, 59614.

[2] N. Bai, L. Wang, Y. Xue, Y. Wang, X. Hou, G. Li, Y. Zhang, M. Cai, L. Zhao, F. Guan, ACS nano 2022.
